# Supplementary material for: Nano-biosupercapacitors enable autarkic sensor operation in blood
Source: Nat Commun. 2021 Aug 23;12:4967. doi: 10.1038/s41467-021-24863-6 (PMC8382768; doi:10.1038/s41467-021-24863-6)
Supplement: Supplementary file 1 — Supplementary Information: Supplementary Figures 1-38; Notes 1-10; Table 1; [file 41467_2021_24863_MOESM1_ESM.pdf]

Supplementary information

Nano-Biosupercapacitors Enable Autarkic Sensor Operation in Blood

Yeji Lee[1,2,3], Vineeth Kumar Bandari[1,2,3]*, Zhe Li[1,2,3], Mariana Medina-Sánchez[3], Manfred F. Maitz[4], Daniil Karnaushenko[3], Mikhail V. Tsurkan[4], Dmitriy D. Karnaushenko[3], & Oliver G. Schmidt[1,2,3,5]*

[1]*Material Systems for Nanoelectronics, Chemnitz University of Technology, 09107 Chemnitz, Germany.*

[2]*Research Center for Materials, Architectures and Integration of Nanomembranes (MAIN), Chemnitz University of Technology, 09126 Chemnitz, Germany.*

[3]*Institute for Integrative Nanosciences, Leibniz IFW Dresden, 01069 Dresden, Germany.*

[4]*Leibniz-Institut für Polymerforschung Dresden e.V., 01069 Dresden, Germany.*

[5]*Nanophysics, Faculty of Physics, TU Dresden, 01062 Dresden, Germany.*

*Correspondence and requests for materials should be addressed to O.G.S. (email: oliver.schmidt@main.tu-chemnitz.de) or to V.K.B. (email: vineeth-kumar.bandari@main.tu-chemnitz.de)

The PDF file includes:
Supplementary Figures 1-38;
Supplementary Notes 1-10;
Supplementary Table 1;
Supplementary References.

Other Supplementary Materials for this manuscript include the following:

Supplementary Movie 1. Effect of lacking passivation on planar nBSC.
Supplementary Movie 2. Effect of multiple GCD cycling on blood plasma plus 0.5% redox dye.
Supplementary Movie 3. Experimental setup – blood flow profile.
Supplementary Movie 4. Blood Flow Profile at 0.05 mms-1.
Supplementary Movie 5. Blood Flow Profile at 0.5 mms-1.
Supplementary Movie 6. Blood Flow Profile at 1 mms-1.
Supplementary Movie 7. Simulation of blood flow velocity profile as a function of nBSC tube diameter.
Supplementary Movie 8. Simulation of blood flow velocity profile as a function of nBSC wall thickness.

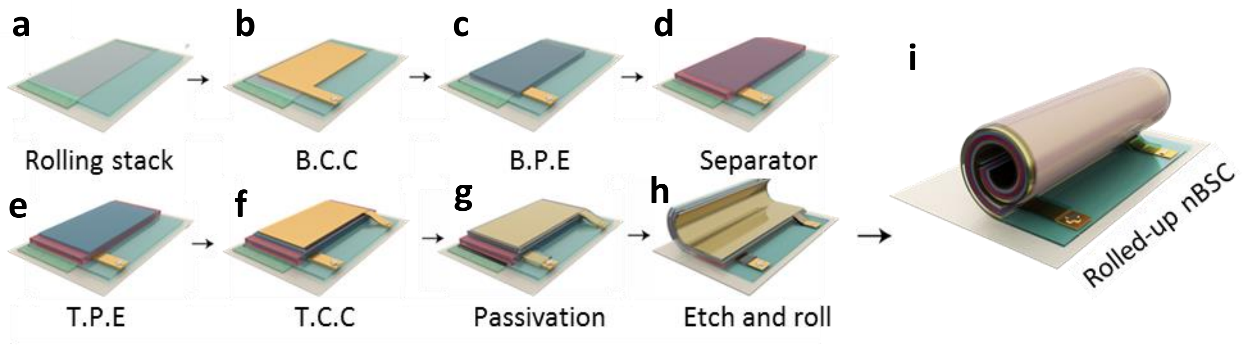

Supplementary Figure 1 | Schematic illustration of nBSC fabrication. a, Strained polymeric rolling stack[1,2] . **b,** Bottom current collector (B.C.C), Cr/Au. **c,** Bottom PEDOT:PSS electrode (B.P.E). **d,** Proton exchange separator, photo-patterned PVA. **e,** Top PEDOT:PSS electrode (T.P.E). **f,** Top current collector (T.C.C), Cr/Au. **g,** SU8 photoresist passivation for quasi-electrical and ionic isolation. **h,** Etching and rolling procedure. **i,** Final rolled-up nBSC.

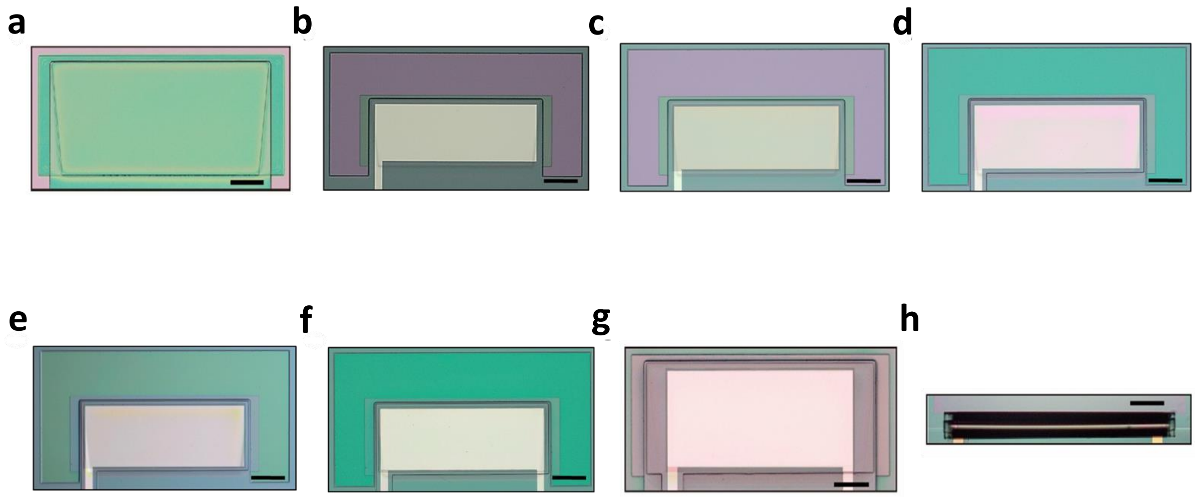

Supplementary Figure 2 | Optical images of a nBSC at consecutive fabrication steps. a, Strained polymeric rolling stack[1,2]. **b,** Bottom current collector, Cr/Au. **c,** Bottom electrode, PEDOT:PSS. **d,** Proton exchange separator, photo-patterned PVA. **e,** Top electrode, PEDOT:PSS. **f,** Top current collector, Cr/Au. **g,** Completed flat nBSC with SU8 photoresist passivation. **h,** Final rolled-up nBSC. Scale bar, 200 µm **(a-h).**

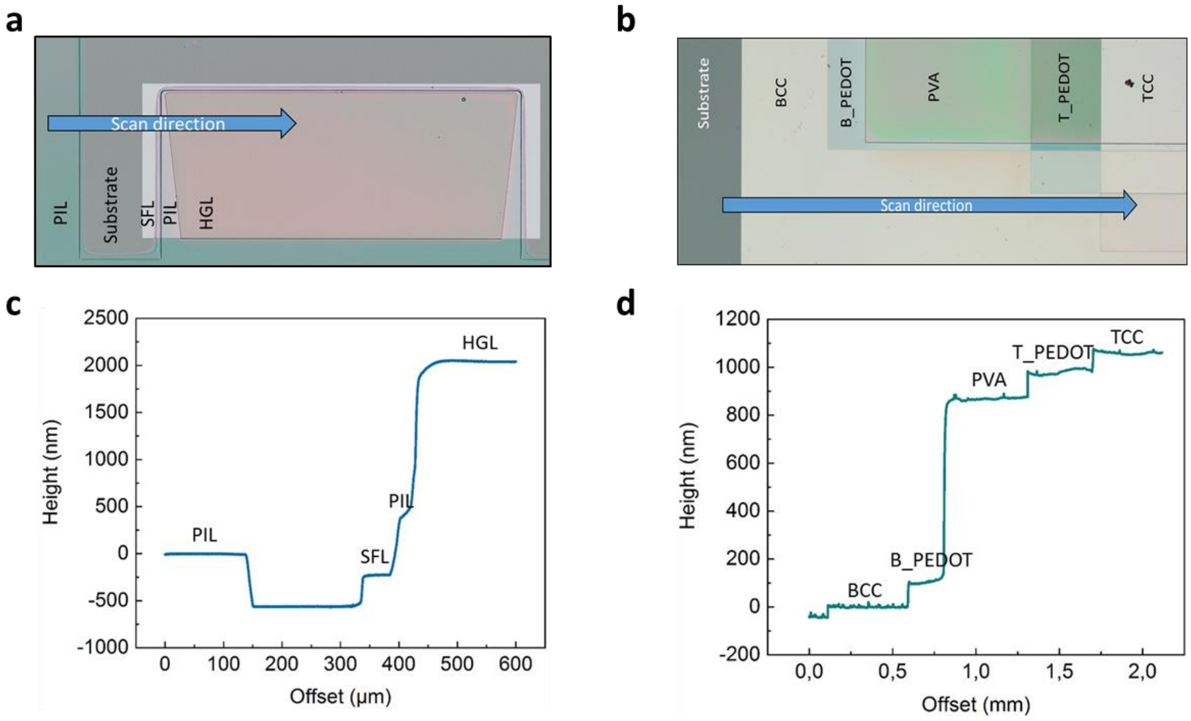

Supplementary Figure 3 | Thickness analysis of material layers of a nBSC. a, Microscope image of strained polymeric rolling stack. **b,** Microscope image of working layer stack of flat nBSC. **c,** Thickness variation across strained polymeric rolling stack; sacrificial layer (SFL), hydrogel layer (HGL), polyimide layer (PIL). **d,** Thickness of flat nBSC; bottom current collector (BCC), bottom PEDOT electrode (B_PEDOT), proton exchange PVA separator (PVA), top PEDOT electrode (T_PEDOT), top current collector (TCC).

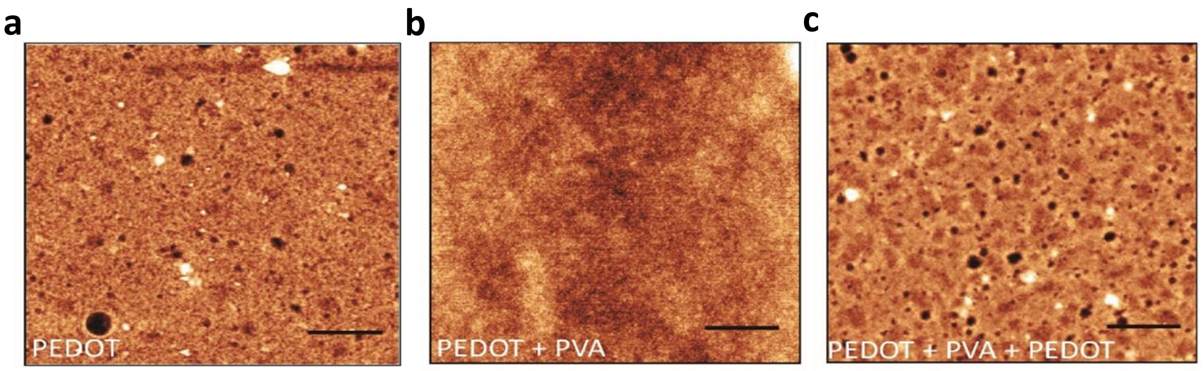

Supplementary Figure 4 | AFM analysis of working electrode material layers. a, 100 nm-thick PEDOT layer on pure silicon substrate. **b,** Bilayer stack with 100/500 nm-thick PEDOT/PVA, respectively **c,** Trilayer stack with 100/500/100 nm-thick PEDOT/PVA/PEDOT, respectively. Scale bar, 2 μm **(a-c)**.

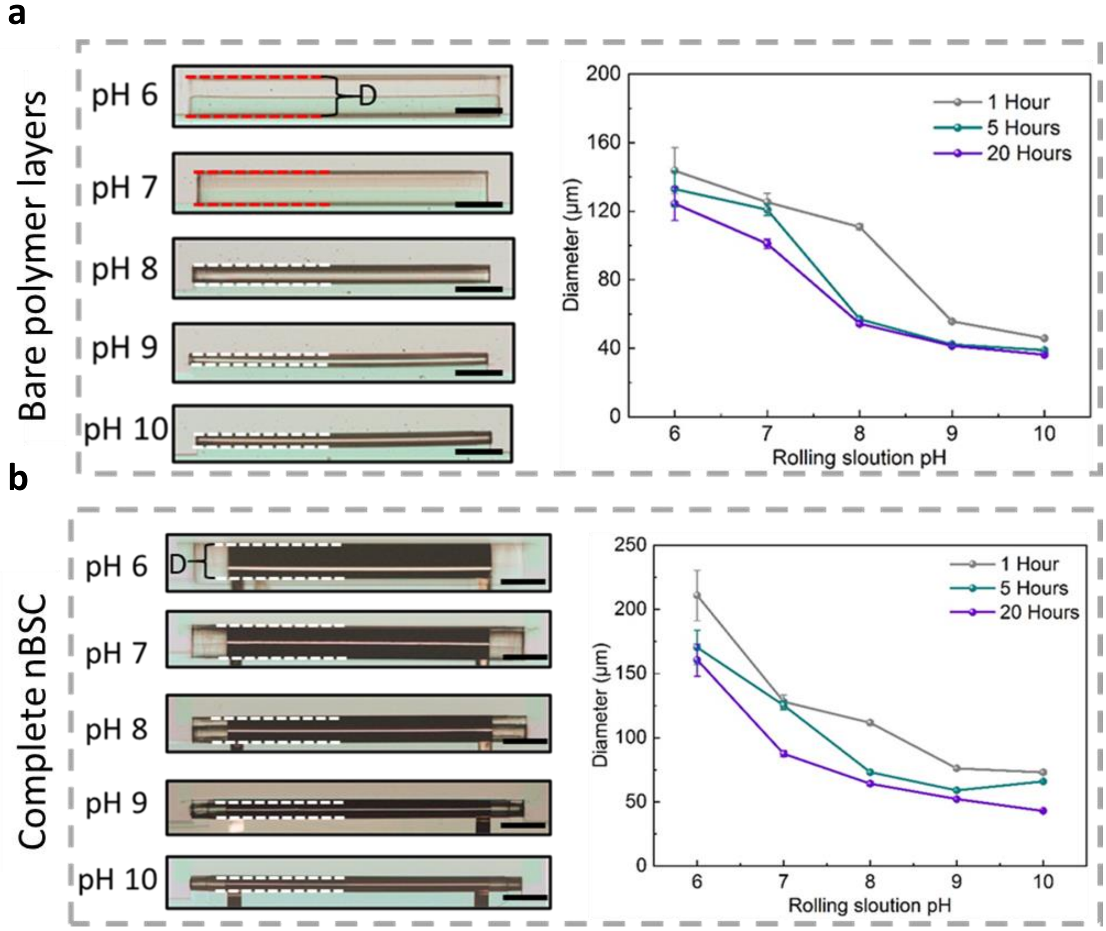

Supplementary Figure 5 | Effect of rolling solution pH on nBSC diameter. a, (left) Top-view optical images of bare polymer tubes rolled in different pH solutions after 20h. (right) Tube diameter as a function of rolling solution pH and time of rolling. **b,** (left) Top-view optical images of "Swiss-roll" nBSCs in different pH solutions after 20h. (right) Tube diameter as a function of rolling solution pH and time of rolling. Scale bar, 150 µm **(a-b)**. Error bars represent the variation of tube diameter over fifty measured devices.

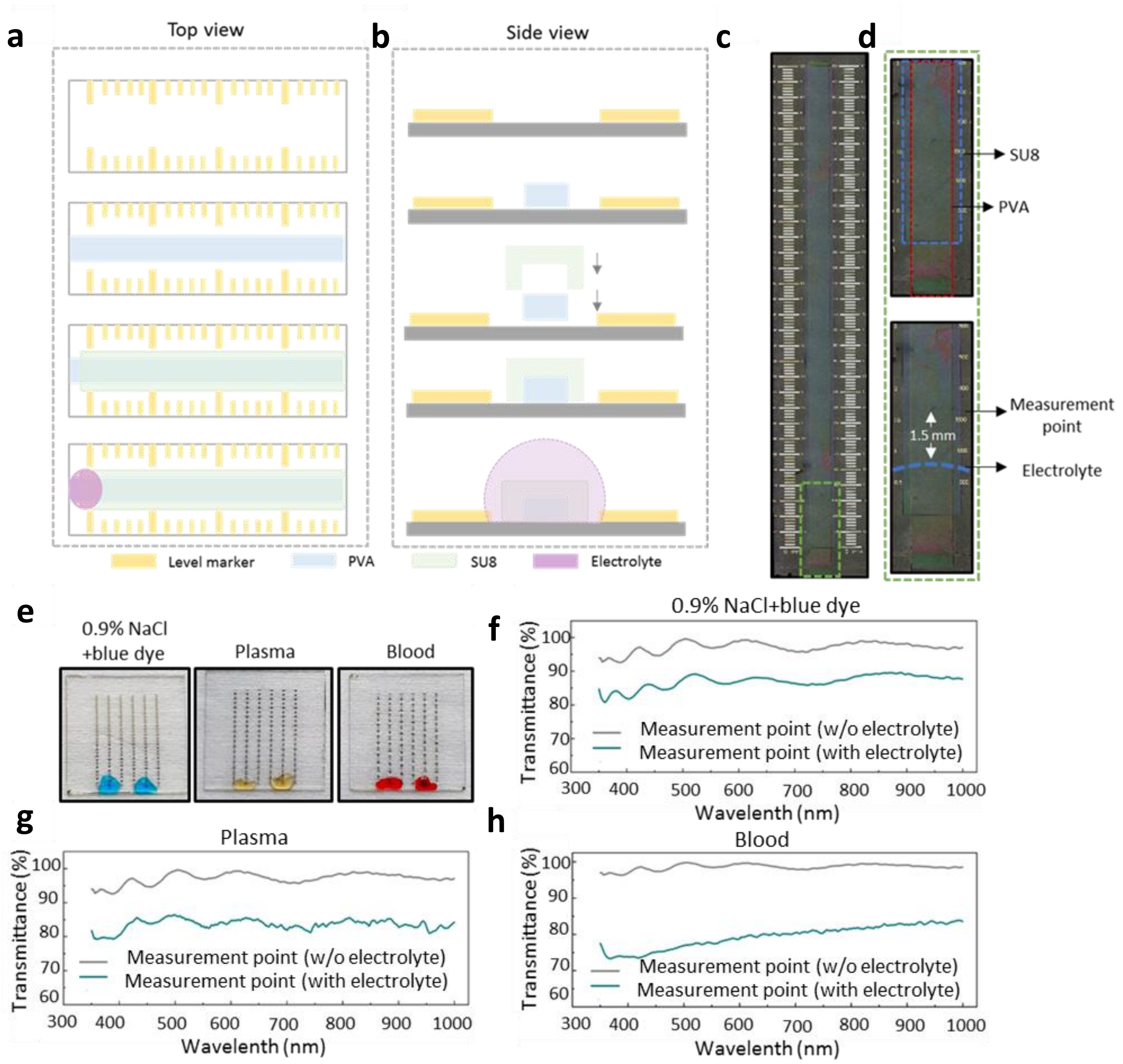

Supplementary Figure 6 | Ability of PVA separator to absorb electrolyte. a-b Schematic illustrating the fabrication of PVA-SU8 stripe for electrolyte absorption test. **(a)** top view and **(b)** side view. **c,** Microscope image of PVA-SU8 stripe for transmittance and absorbance spectroscopy. **d,** (top) Magnified image of PVA-SU8 stripe showing individual components. (bottom) Electrolyte drop position with three measurement points on PVA-SU8 stripe. **e,** Optical image of PVA-SU8 separator stripe with blue dye-NaCl electrolyte (left), PVA-SU8 separator stripe with blood plasma electrolyte (center) and PVA-SU8 separator stripe with blood electrolyte (right). **f,** Transmittance spectrum through PVA-SU8 stripe with absorbed blue dye-NaCl electrolyte. **g,** Transmittance spectrum through PVA-SU8 stripe with absorbed blood plasma electrolyte. **h,** Transmittance spectrum through PVA-SU8 stripe with absorbed blood electrolyte.

Supplementary Notes

Supplementary Note 1 Ability of PVA separator to absorb electrolyte

Despite the coverage by the current collectors, PEDOT electrodes can well interact with redox enzymes and plasma due to the presence of the PVA separator. PVA is a porous hydrogel matrix that efficiently absorbs and sucks in the electrolyte and its components at the edge of the Swiss-roll nBSC device[3]. As the PEDOT electrode surface is in tight contact with the PVA separator, the redox enzymes in the blood can interact with the entire surface of the PEDOT, thus leading to significant performance enhancement. In order to estimate the absorption of the PVA separator, PVA-SU8 stripes were prepared on glass substrate as shown in Supplementary: Fig. 6a-c (see Supplementary: Note 1 for detailed fabrication process). These PVA-SU8 stripes were used to perform transmittance spectroscopy before and after dropping the three different electrolytes (0.9% NaCl, blood plasma and blood) at one end of the PVA-SU8 stripes. After allowing the liquid to diffuse for a few minutes, measurements were performed at a distance of 1.5 mm away from the electrolyte drop edge (see Supplementary: Fig. 6c-d) which is way beyond the full length of the nBSC (~1 mm). As shown in Supplementary: Fig. 6f-h, after the introduction of the respective electrolytes (for optical absorption properties of electrolytes, see e.g. 0.9% NaCl[4,5], blood plasma[6-8] and blood[9,10]) the transmittance spectra at the measurement point are significantly decreased. This decrease in transmittance before and after introduction of the electrolyte shows that the electrolyte and all its components (redox enzymes and other components) diffuse through the PVA hydrogel separator and can thus provide efficient interaction with the PEDOT electrode surface.

Sample preparation

Step 1: *Level markers:* Standard photolithography was used to negatively pattern AZ 5214E photoresist (from Micro Chemicals) on the polymeric layer stack. After patterning the photoresist, 10 nm Cr and 50 nm Au were deposited at 0.5 Å s^{-1} in an e-beam evaporator (Creavac). Finally, a lift-off process was performed in acetone and isopropyl alcohol to remove the photoresist and residue metal layer.

Step 2: *PVA hydrogel separator:* The separator membrane was synthesised by mixing 1g poly (vinyl alcohol) (PVA, from Sigma Aldrich) and 5 mg potassium dichromate in 10 ml DI-Water at 80°C for 12 hours. The solution was cooled and spin coated at 9,000 RPM for 60 s by using a 0.45 µm PVDF filter and soft baked at 40°C for 4 mins to deposit a ~500 nm thick film. The PVA films were then photo-exposed (365 nm, 15 Wcm^{-2}) for 90 s through a photomask and the separator was patterned by developing the samples in DI water for 20 s (The topography can be shown in Supplementary: Fig. 4).

Step 3: *SU8 photoresist Passivation:* A ~600 nm thick SU8-2000.5 (from Micro resist technology) was spin-coated at 4,500 RPM for 60 s and soft baked at 95°C for 4 mins. The samples where then patterned to remove the SU-8 to form a trench for rolling by UV-exposure (365 nm, 15 Wcm^{-2}) for 65 s through a photomask and a post exposure bake at 95°C for 2 mins was done to accelerate the crosslinking process of the exposed area. Finally, the trench was formed by developing the samples in mr-Dev 600 (from Micro resist technology) for 60 s.

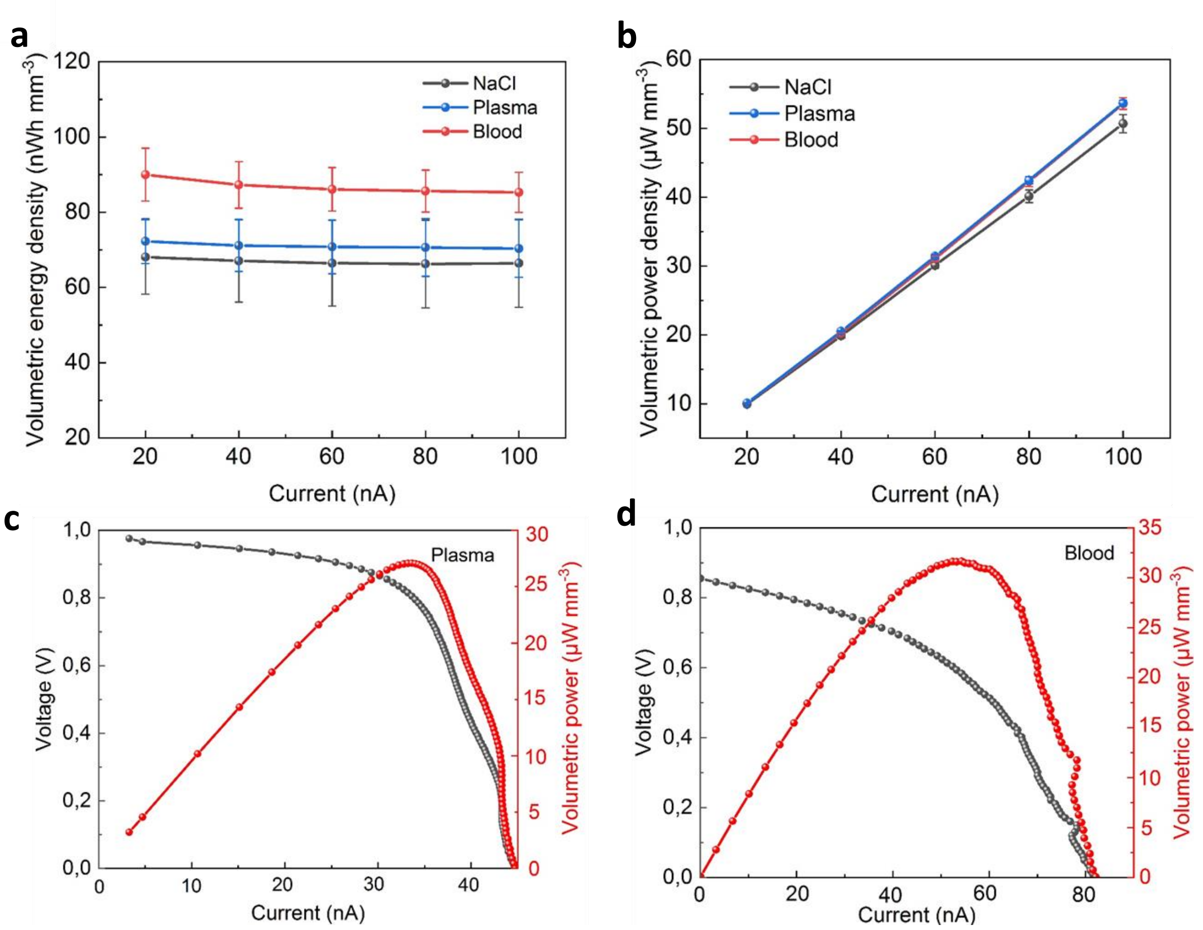

Supplementary Figure 7 | Volumetric energy and power densities of nBSCs in three different electrolytes. a, Energy density and **b,** Power density as a function of applied currents (20, 40, 60, 80 and 100 nA) per unit volume in 0.9% NaCl, blood plasma and blood electrolyte. (all measurements were performed at room temperature (25 °C) and static flow (0 ml/min). Error bars represent the variation in data of three measured devices.) **c-d,** Current-voltage and current-power curves of nBSC in **(c)** blood plasma and **(d)** blood electrolyte.

Supplementary Note 2 Electrochemical characterization

The electrochemical performance of planar/tubular nBSCs was characterized by cyclic voltammetry (CV), galvanostatic charge-discharge (GCD), self-discharge, and electrochemical impedance spectroscopy (EIS) using the μAutolab Type III potentiostat from Metrohm. The volumetric specific capacitance (C_V, F mm^{-3}) of the device was calculated from GCD curves according to Equation S1:

$$C_V = \frac{I}{V} \times \frac{\Delta t}{\Delta V} \quad (1)$$

where volume (V) of the 3D tubular nBSCs, ΔV is the difference between the initial potential (V_0) and final potential (V_1), I is the applied constant current (A), and Δt is the discharge time (t_0-t_1).

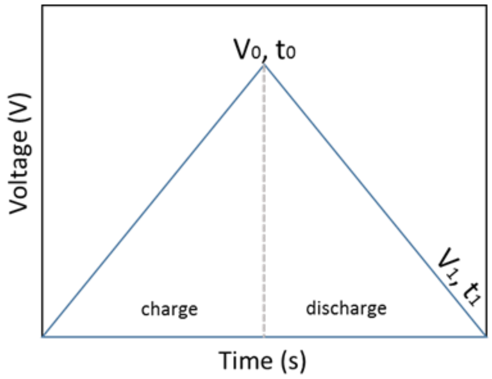

The volumetric energy density (E_V, Wh mm^{-3}) and power density (P_V, W mm^{-3}) were calculated according to Equation S2 and S3:

$$E_V = \frac{1}{2} \times C_V \times \frac{(\Delta V)^2}{3600} \quad (2)$$

$$P_V = \frac{E_V}{\Delta t} \times 3600 \quad (3)$$

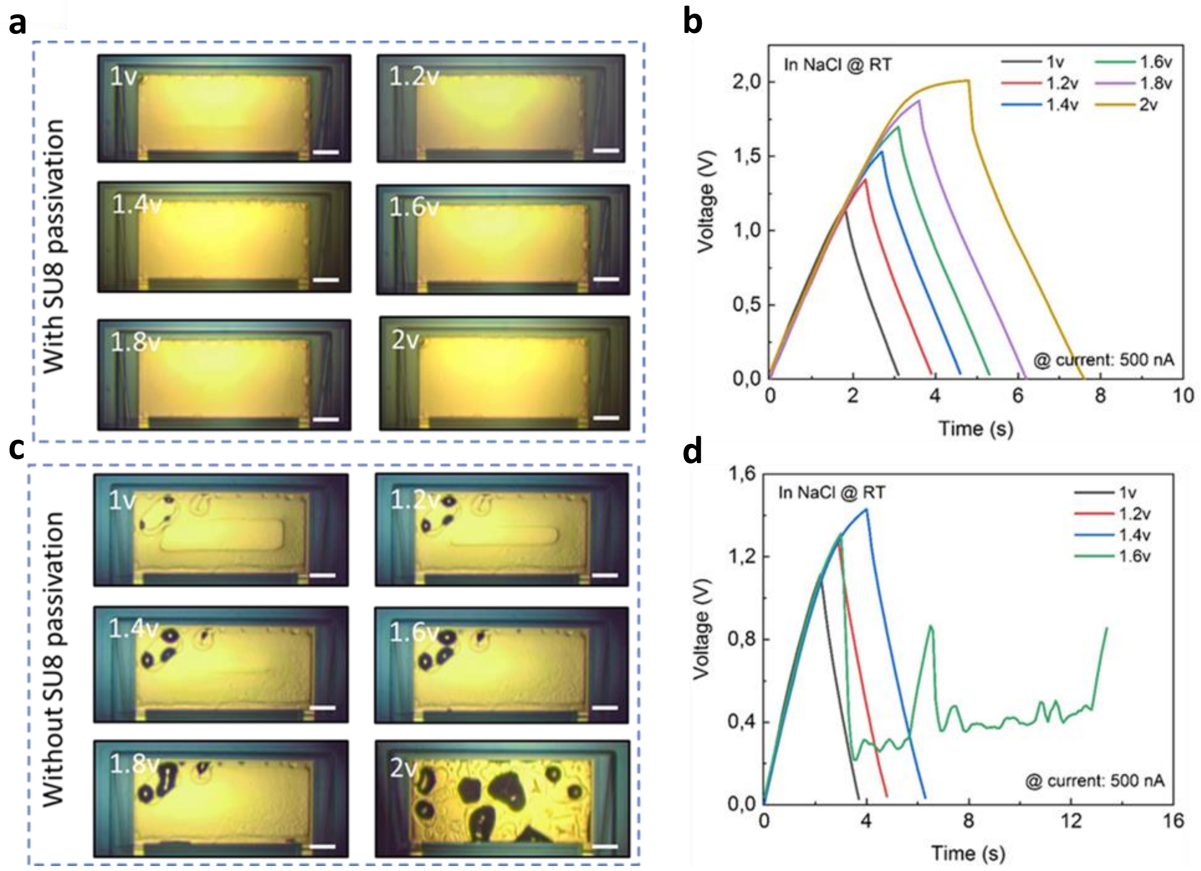

Supplementary Figure 8 | Effect of SU8 photoresist passivation on planar nBSC. a, Optical images of stable SU8 photoresist passivated device in 0.9% NaCl electrolyte after charging to different voltages (1, 1.2, 1.4, 1.6, 1.8 and 2 V). **b,** GCD curves of nBSC charged up to 2 V at 500 nA. **c,** Optical images of unstable non-passivated device in 0.9% NaCl electrolyte after charging to different voltages (1, 1.2, 1.4, 1.6, 1.8 and 2 V). **d,** GCD curves of nBSC charged up to 1.6 V at 500 nA. Scale bar, 150 µm.

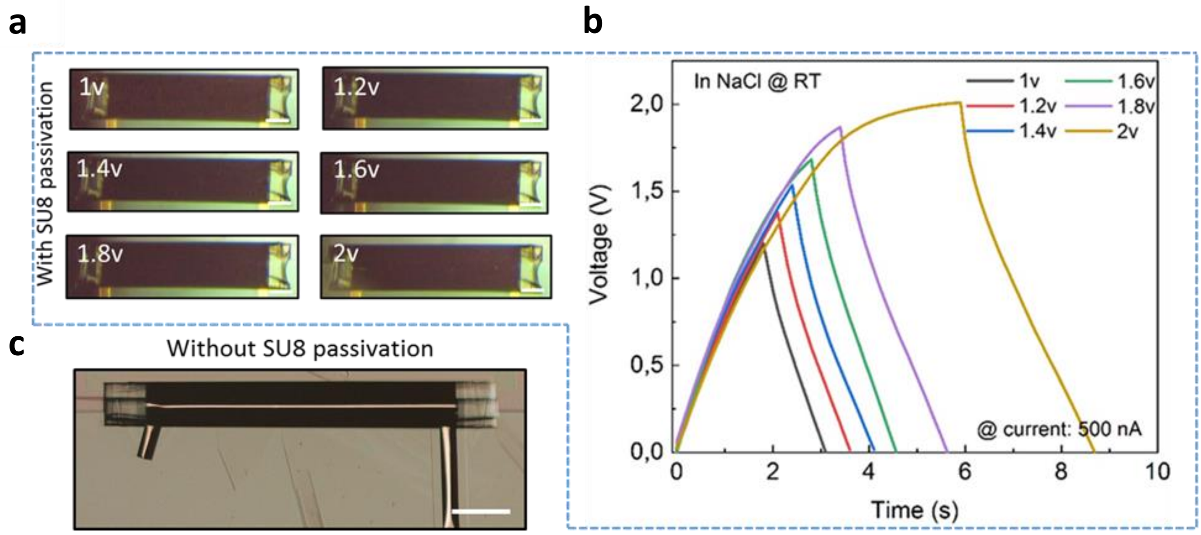

Supplementary Figure 9 | Effect of SU8 photoresist passivation on "Swiss-roll" nBSC. a, Optical images of stable SU8 photoresist passivated "Swiss-roll" nBSC in 0.9% NaCl electrolyte after charging to different voltages (1, 1.2, 1.4, 1.6, 1.8 and 2 V). **b,** GCD curves of nBSC charged up to 2 V at 500 nA. **c,** Optical images of unstable non-passivated "Swiss-roll" nBSC. Scale bar, 150 µm.

Supplementary Note 3 Effect of SU8 photoresist passivation on nBSCs

As the nBSC performs in liquid electrolyte such as 0.9% NaCl, blood plasma and blood and enables charging up to 1.6 V, it raises concerns about how the device can operate at 3 V without any gas evolution or water splitting. To suppress water splitting and enable stable operation, nBSCs were passivated with a 500 nm thick insulating SU8 photoresist layer isolating the system electrically and ionically. This quasi-electronic and ionic isolation significantly reduces the gas evolution and water-splitting reaction. As shown in Supplementary: Fig.8a-b and 9a-b, the devices with SU8 photoresist passivation (before and after rolling) show no signs of degradation, gas evaluation or water splitting. In contrast, the non-passivated devices show significant degradation when charged to 1.6 V and significant gas evolution due to water splitting is observed (see Supplementary: Fig. 8c-d and Movie 1). Moreover, the non-passivated devices were also damaged during the rolling process yielding a non-functional device (see Supplementary: Fig. 9c).

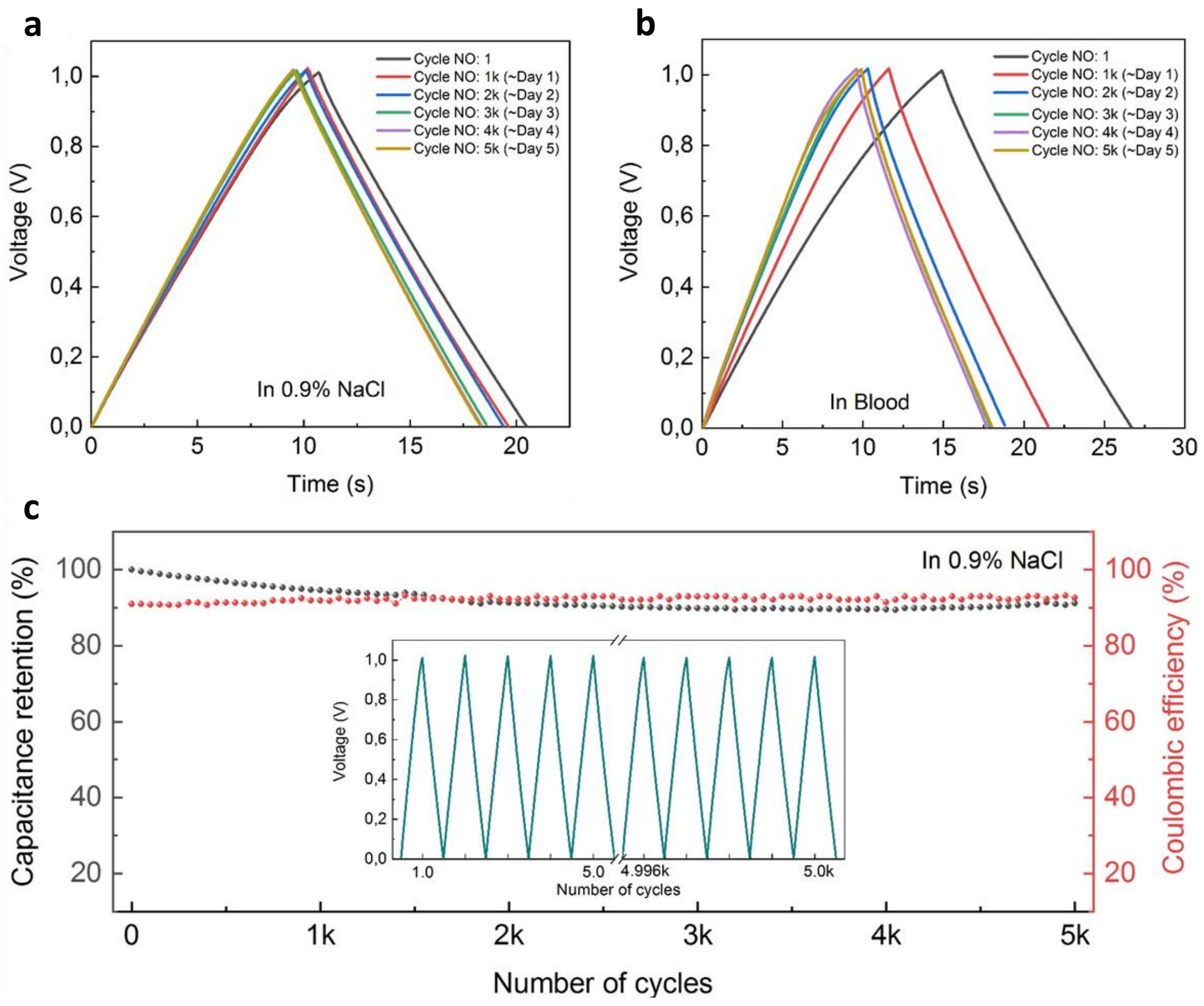

Supplementary Figure 10 | Capacitance retention of nBSCs over 5000 cycles in different electrolytes. a-b, GCD curves of tubular nBSC for every 1000 cycles extracted from multiple cycling test (5000x cycles) at 50 nA in **(a)** 0.9% NaCl and **(b)** blood electrolyte. **c,** Capacitance retention and coulombic efficiency in 0.9% NaCl over 5000 cycles. (all measurements were performed at room temperature (25 °C) and static flow (0 ml/min).)

Supplementary Note 4 Effect of electrolyte stability on capacitance retention of nBSCs

The lifetime of nBSCs was investigated by multiple charge-discharge (GCD) cycles over 5000x in blood electrolyte. As shown in Fig. 1j, the device experienced a 30% decay of capacitance retention over 5.5 days. The decay of capacitance results from the active decomposition of blood electrolyte. The measurements were performed in a petri-dish under standard ambient conditions (without applying oxygen, carbon dioxide, humidity and glucose). Such a condition results in gradual cellular apoptosis and decomposition of biological enzymes decreasing bioelectrocatalytic activity and eventually stopping any bioenhancement behaviour. The control measurement (5000x GCD cycles) in 0.9% NaCl confirms this interpretation. As shown in Supplementary: Fig. 10a and 10c, the device shows excellent cycling stability and high capacitance retention over ~90%. The NaCl is a non-biological fluid and does not decompose over the measurement period (5.5 days).

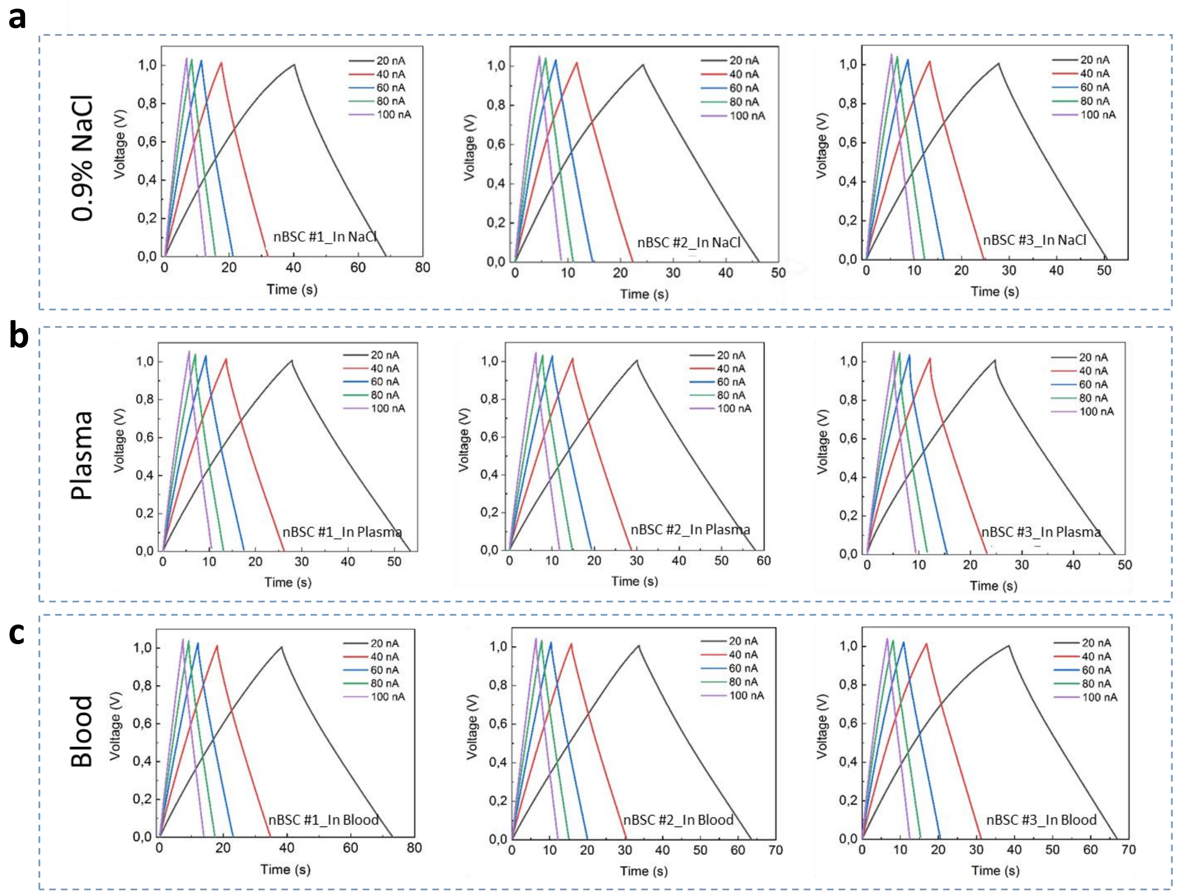

Supplementary Figure 11 | GCD measurements of nBSCs in three working electrolytes. a-c, GCD curves of three different devices at 20, 40, 60, 80 and 100 nA in **(a)** 0.9% NaCl, **(b)** blood plasma and **(c)** blood electrolyte. (all measurements were performed at room temperature (25 °C) and static flow (0 ml/min).)

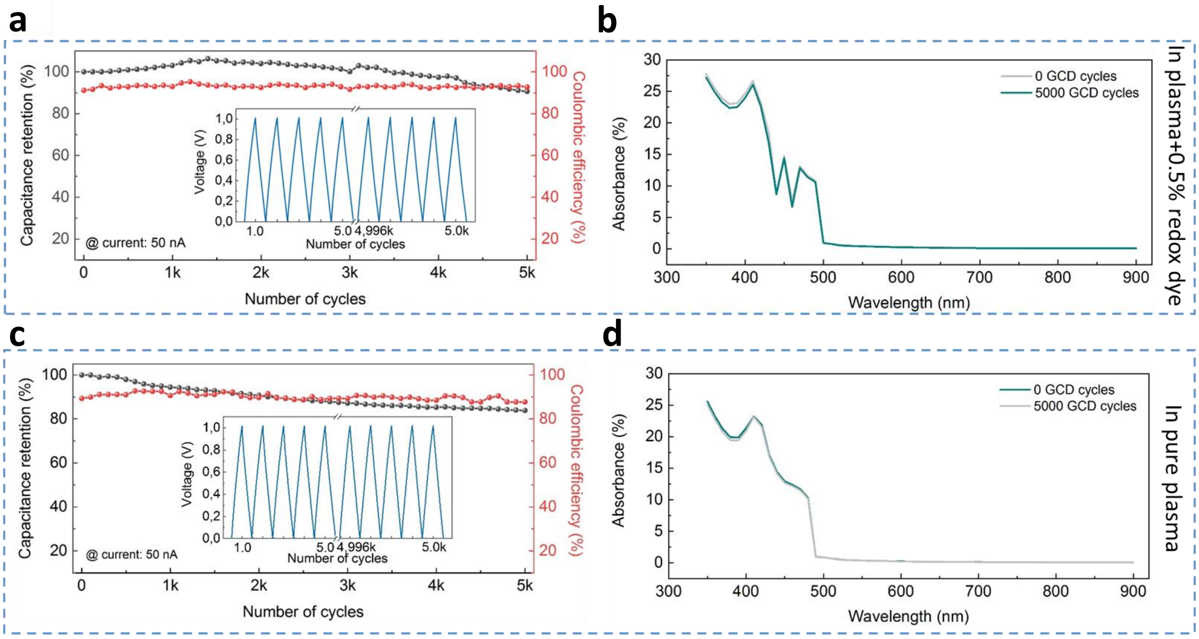

Supplementary Figure 12 | Effect of multiple GCD cycling on blood plasma chemical compositions. a, Capacitance retention and coulombic efficiency of a device in blood plasma including 0.5% redox dye over 5000x GCD cycles. Inset shows GCD curves at 50 nA. **b,** Spectrophotometric characterization of blood plasma treated with 0.5% redox dye before and after 5000x GCD cycles. **c,** Capacitance retention and coulombic efficiency of a device in blood plasma over 5000x GCD cycles. Inset shows GCD curves at 50 nA. **d,** Spectrophotometric characterization of blood plasma before and after 5000x GCD cycles.

Supplementary Note 5 Effect of multiple GCD cycling on blood plasma chemical compositions

The human body typically accommodates 5 liters of blood, which constantly circulates through the body[11,12]. This blood is reused by the body several times and undergoes constant replenishment through efficient recycling and detoxification processes present in our kidney. The introduction of a nBSC into a blood vessel would not significantly consume redox enzymes and other chemicals from the blood, as a simple estimation shows that the hydrogel layer and the PVA separator in the nBSC absorb approximately ~0.5 nl of electrolyte during a full blood circulation. This volume is 10 billion times smaller than the total blood volume in an average human body and would not cause any health risk[13-16]. Nevertheless, in order to check out any change in chemical composition of biological electrolytic fluid, nBSCs integrated into a PDMS microfluidic channel were fabricated and 5 ml of two different electrolyte system: pure blood plasma and blood plasma mixed with 0.5% methyl viologen dichloride hydrate (redox dye) were prepared. The redox dye was introduced to blood plasma in order to visualize the active reduction of electrolyte during the charging phase of the nBSC. After the electrolyte preparation, 400 µl of both electrolytes were separately collected as a reference for absorption spectrophotometric characterization. Following this, the microfluidic channel was connected to a syringe pump generating a forth-and-back flow rate of 100 µl min^{-1} of the remaining blood plasma redox electrolytes. And 5000x galvanostatic charge-discharge (GCD) cycling were performed of the nBSC device (see Supplementary: Fig. 12a, Movie 2) and finally a 400 µl sample of the electrolyte was collected at the end of the 5000x cycles for absorption spectrophotometric characterization. As compared in Supplementary: Fig. 12b and Movie 2, the redox dye in blood plasma does not show any evident change in absorption spectrum or color of the blood plasma electrolyte during charging or discharging. In order to confirm this, 5000x GCD cycles were performed on the pure blood plasma system as a control. As shown in Supplementary: Fig. 12c-d, the absorption spectrum of the blood plasma collected after 5000x GCD cycles does not show any significant deviation compared to the reference before cycling, either. This result combined with the large volume of blood and natural recycling system present in a typical human body shows that the nBSCs would not pose any health risks as there would be no significant changes to the chemical compositions of these fluids.

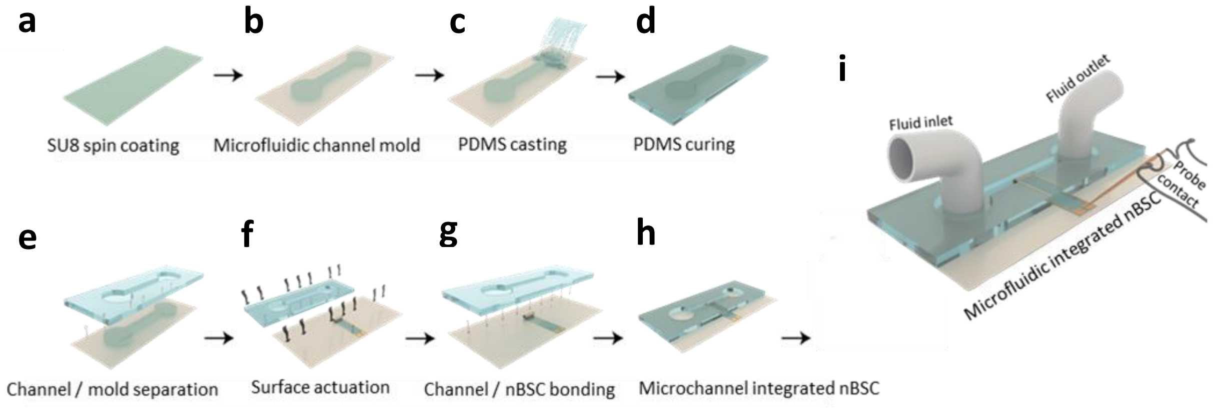

Supplementary Figure 13 | Schematic illustration of nBSC fabrication and integration into a microfluidic channel. a, 250μm thick spin coated SU8 photoresist layer on Si/SiO_2. **b,** Patterned microfluidic channel mold with UV exposure. **c,** PDMS casting on the micro fluidic SU8 photoresist mold. **d,** PDMS curing around SU8 photoresist mold. **e,** Removal of PDMS microfluidic channel from the mold. **f,** Surface actuation of nBSC chip and PDMS microfluidic channel. **g,** Mechanical bonding of PDMS microfluidic channel with nBSC chip. **h**, Complete integration of micro fluidic channel with nBSC. **i,** Schematic illustration of micro fluidic channel integrated nBSC with inlet and outlet flow and physical contact to measurement platform via micro probes.

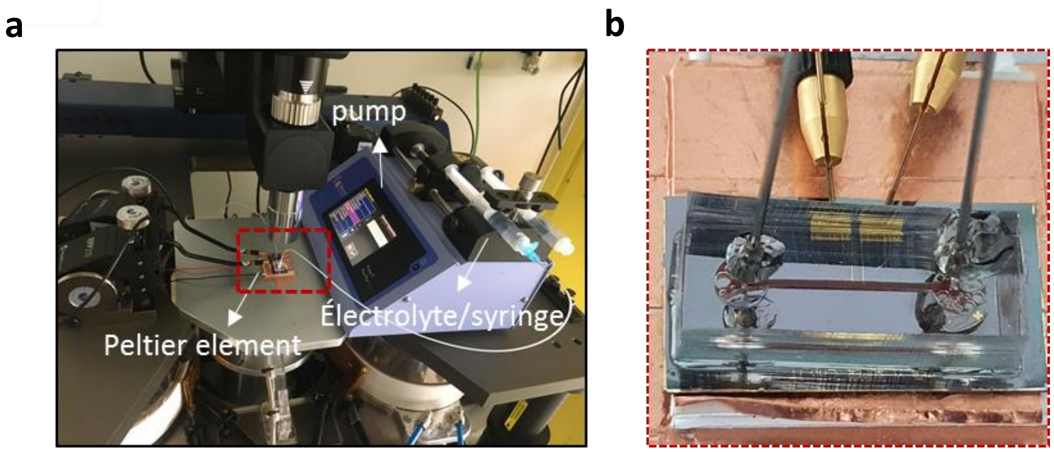

Supplementary Figure 14 | Flow dependent electrochemical measurement setup. a, Measurement setup for flow dependent analysis. **b,** Optical image of nBSC integrated into a PDMS micro fluidic channel with micro probes connected to the bond pads for electrochemical analysis.

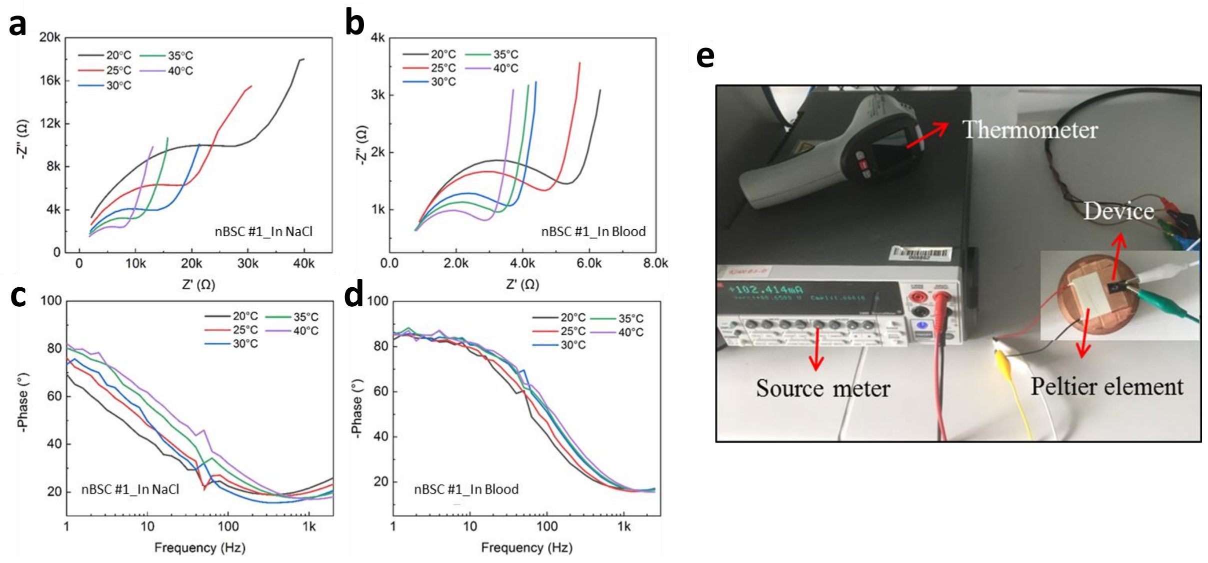

Supplementary Figure 15 | nBSC impedance as a function of temperature. a-b, Impedance spectra at different temperatures (20, 25, 30, 35, and 40°C) in **(a)** 0.9% NaCl and **(b)** blood. **c-d,** Impedance phase angle vs. frequency at different temperatures in **(c)** 0.9% NaCl and **(d)** blood. **e,** Measurement setup for temperature dependent analysis. (all measurements were performed at static flow (0 ml/min).)

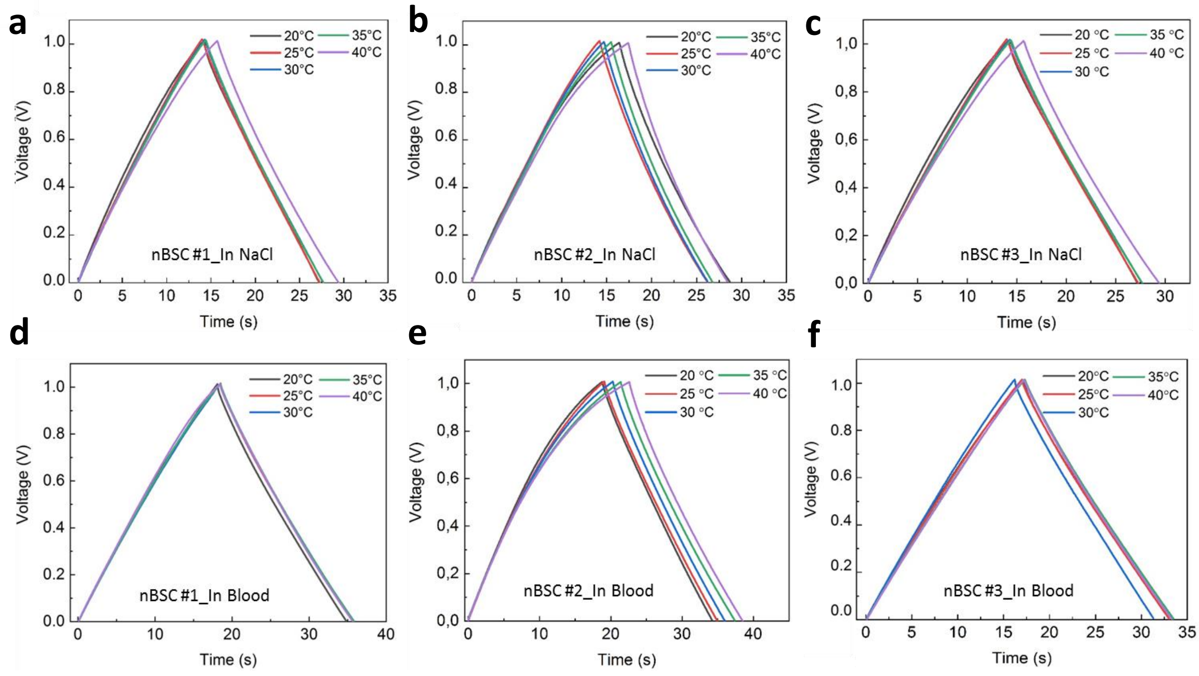

Supplementary Figure 16 | Temperature dependent GCD measurements of nBSCs. a-f, GCD curves at different temperatures (20, 25, 30, 35, and 40°C) at an applied current of 50 nA in **(a-c)** 0.9% NaCl electrolyte and **(d-f)** blood electrolyte. (all measurements were performed at static flow (0 ml/min).)

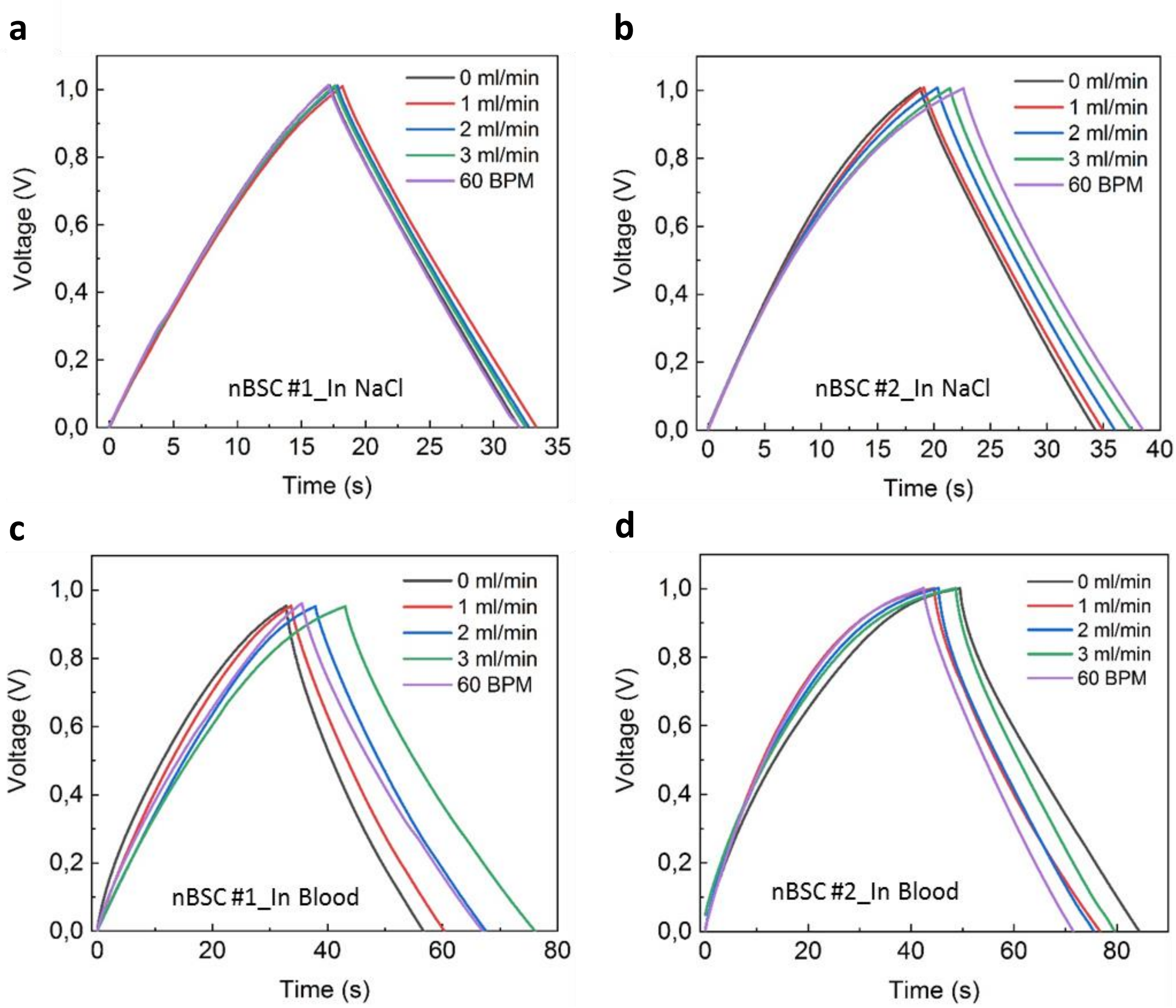

Supplementary Figure 17 | Flow dependent GCD measurements of nBSCs. a-d, GCD curves at different flow rates (static, 1, 2, 3 ml/min and 60 BPM at 3 ml/min) at 50 nA in **(a-b)** 0.9% NaCl and **(c-d)** blood. (all measurements were performed at room temperature (25 °C).)

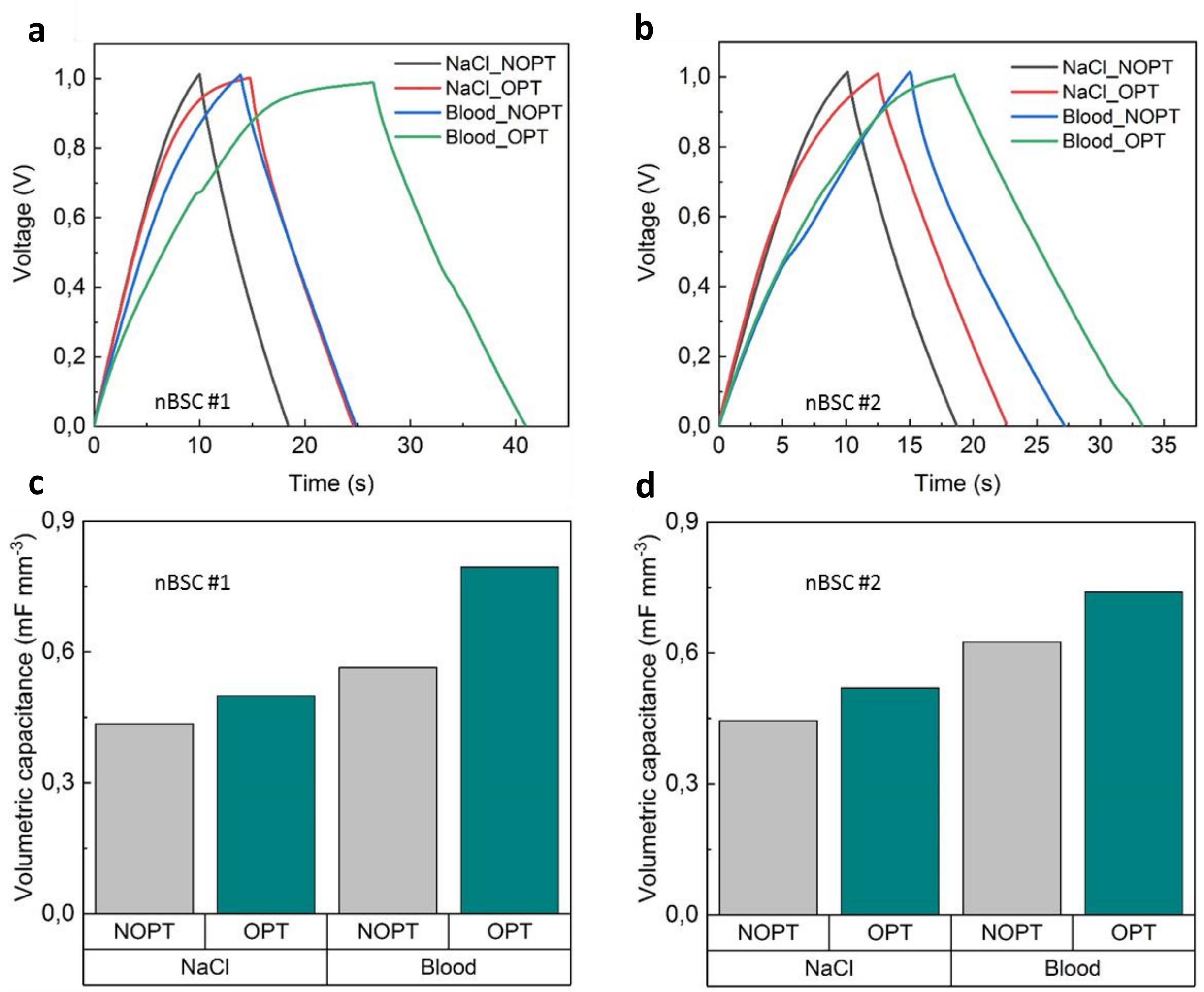

Supplementary Figure 18 | Effect of oxygen plasma treatment. a-b, GCD curves of two different devices with oxygen plasma treatment (OPT) and without oxygen plasma treatment (NOPT) at 50 nA in 0.9% NaCl and blood. **c-d,** Comparing the effect of OPT on volumetric capacitance of nBSC in 0.9% NaCl and blood. (all measurements were performed at room temperature (25 °C) and static flow (0 ml/min).)

Supplementary Note 6 Effect of oxygen plasma treatment on nBSC performance

In order to measure the performance of nBSCs as a function of electrolyte flow, the nBSCs were integrated into a PDMS-based microfluidic channel. This integration requires a few additional fabrication steps such as oxygen plasma treatment (OPT, in PVA-TEPLA at 400W for 1 min using microwave oxygen plasma) to activate the nBSC sample surface and actively bond it to the PDMS-channel. (see method section)

OPT as a plasma treatment process involves the interaction of the sample with highly energetic ions, UV radiation and radicals[17]. These reactions typically generate surface free-radicals, such as superoxide anions ($O^{2}-$), hydroxyl ions ($OH-$), hydrogen peroxide (H_2O_2), nitric oxide (NO), and hydroxyl radicals (•OH)[18]. These free-radicals in turn generate organic radicals in the photoactive HGL and PIL layers and redox PEDOT polymer electrode.

As shown in Fig. 3e, 3f and Supplementary: Fig. 18, the GCD curve shows asymmetric behaviour and an increase in volumetric capacitance (after OPT) due to the enhanced charge transfer at the electrode and electrolyte interface. The enhancement in volumetric capacitance can be attributed to the presence of surface free and organic radicals in the redox polymer[19]. In charge-storage applications, organic radicals usually store one charge per redox-active unit in the redox processes, resulting in high doping levels (0.8-.09 radicals per monomer)[20] of the molecule via electron transfer. These processes improve electron-transfer rates leading to the enhancement of electrochemical performance of the nBSC[21,22].

Although the OPT results in a substantial enhancement of the electrochemical performance of a nBSC by generating organic free-radicals, the OPT cannot be applied for in-vivo applications, because free-radicals are associated with several physiological degradations, such as DNA damage, thrombosis (clothing), inflammation, cell death and apoptosis[23-25]. Therefore, surface-activation by OPT is only used to create and measure the samples for flow-dependent measurements and not used for any the other electrochemical measurements in this work.

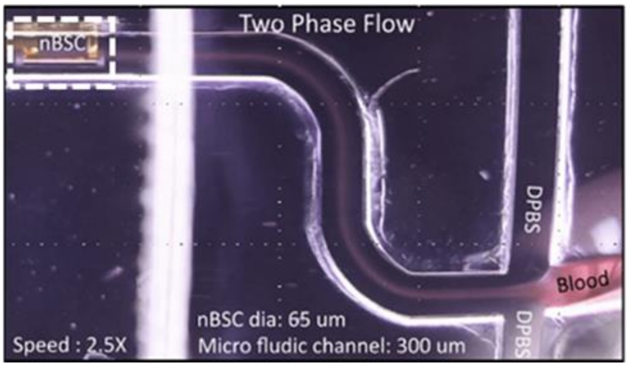

Supplementary Figure 19 | Experimental correlation: nBSC on blood flow profile. Optical image of two-phase flow microfluidic channel measurement setup with phase1: DPBS; Phase2: Blood.

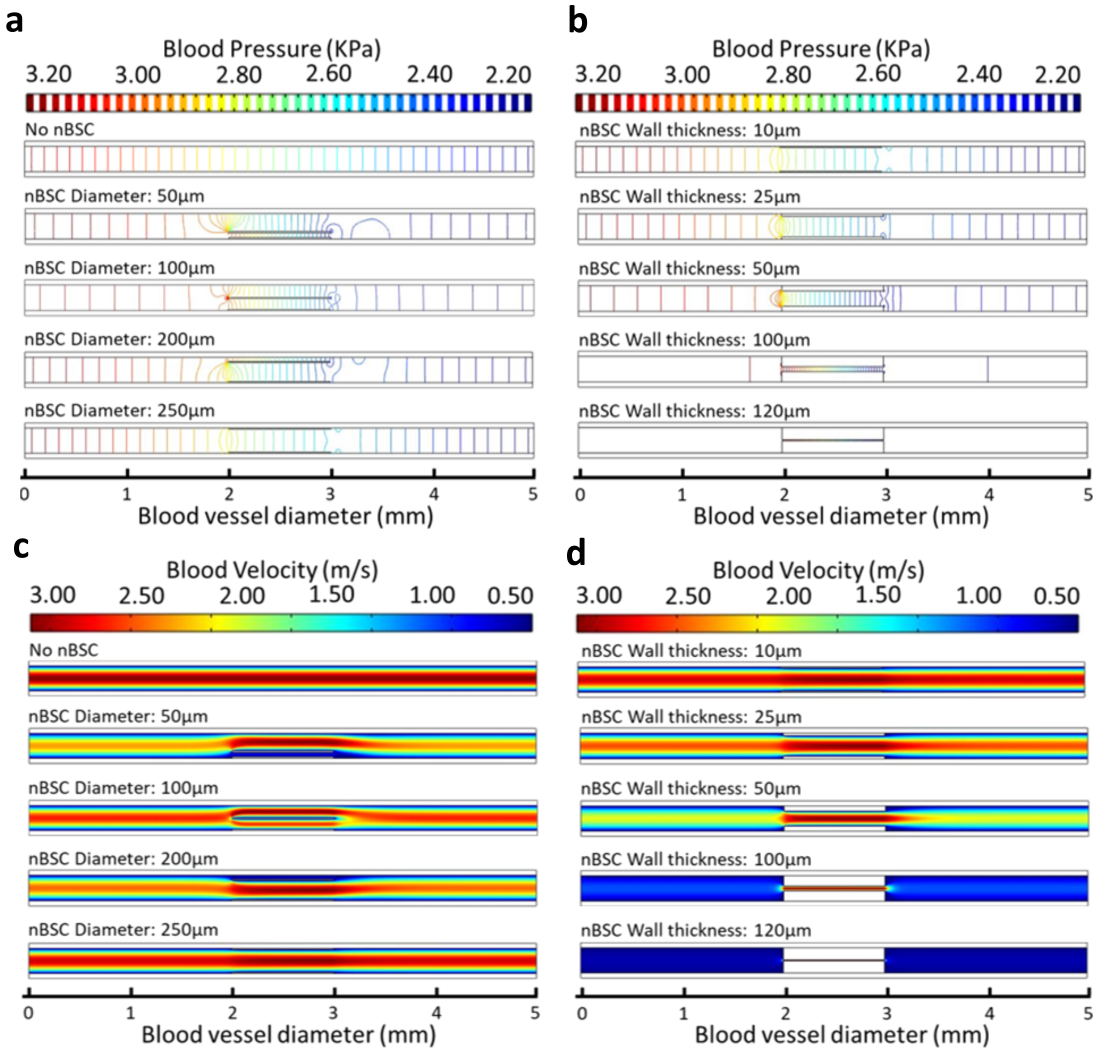

Supplementary Figure 20 | Simulation of blood pressure and flow velocity profile as a function of nBSC outer diameter. a, Pressure profile of the blood vessel as a function of nBSC outer diameter changing from 50 to 250 µm. **b,** Pressure profile of the blood vessel as a function of nBSC inner diameter changing from 10 to 240 µm (which accounts to 96 % to 4 % blockage of blood vessel). **c,** Velocity profile of the blood vessel as a function of nBSC outer diameter changing from 50 to 250 µm. **d,** Velocity profile of the blood vessel as a function of nBSC inner diameter changing from 10 to 240 µm.

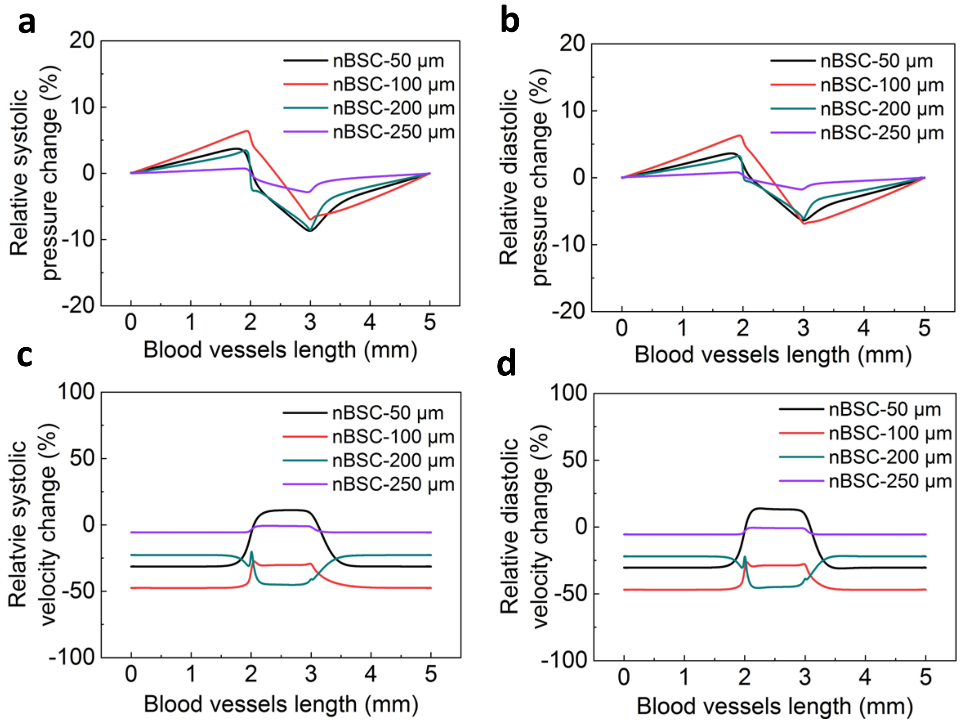

Supplementary Figure 21 | Simulation of blood flow velocity profile as a function of nBSC outer diameter. a-b, Effect of nBSC outer diameter on local blood pressure. **a,** Relative systolic pressure changes along the length of blood vessel as a function of nBSC outer diameter. **b,** Relative diastolic pressure changes along the length of blood vessel as a function of nBSC outer diameter. **c-d,** Effect of nBSC diameter on local blood velocity. **c,** Relative systolic velocity change along the length of blood vessel as a function of nBSC outer diameter. **d,** Relative diastolic velocity change along the length of blood vessel as a function of nBSC outer diameter.

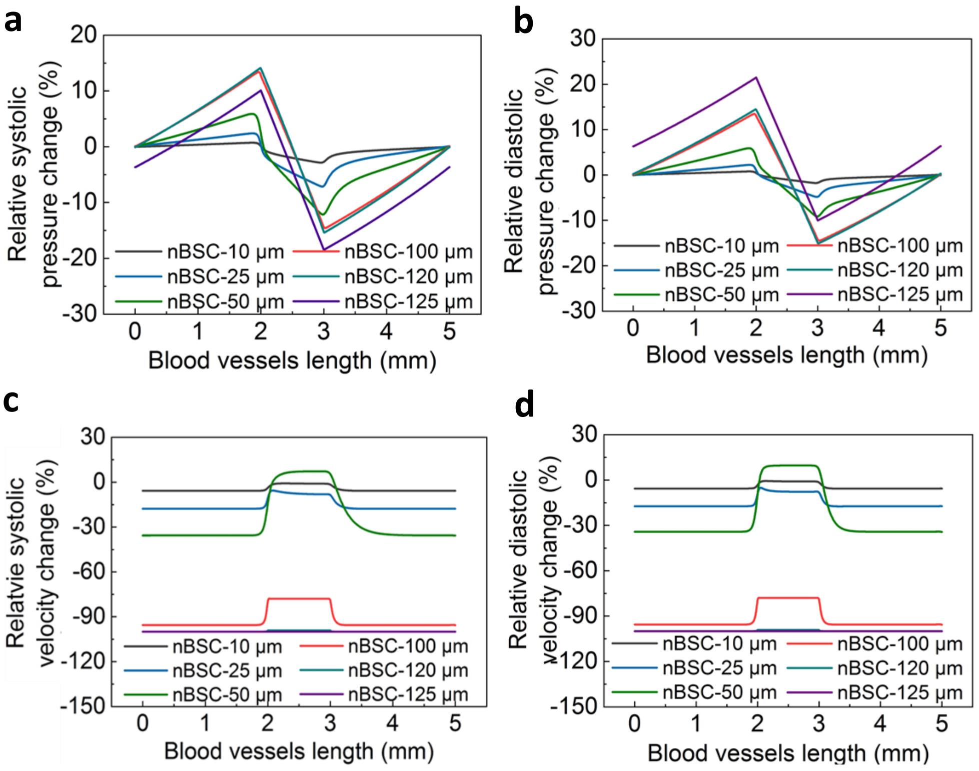

Supplementary Figure 22 | Simulation of blood flow velocity profile as a function of nBSC inner diameter. a-b, Effect of nBSC inner diameter on local blood pressure. **a,** Relative systolic pressure changes along the length of blood vessel as a function of nBSC inner diameter. **b,** Relative diastolic pressure changes along the length of blood vessel as a function of nBSC inner diameter. **c-d,** Effect of nBSC inner diameter on local blood velocity. **c,** Relative systolic velocity change along the length of blood vessel as a function of nBSC inner diameter. **d,** Relative diastolic velocity change along the length of blood vessel as a function of nBSC inner diameter.

Supplementary Note 7 Effect of nBSC on local blood pressure and flow rate

The nBSC device occupies a volume of ~1 to 2 nl (or ~1E-3 to 2E-3 mm-3) which is significantly smaller than many blood vessels (e.g. arteries or veins). Moreover, the hollow core of the tube provides a free-flow channel for blood to pass. Blood vessels have different diameters at various parts of the body, so to understand the blood flow profile through a vessel, flow simulations of a nBSC placed at the wall of the blood vessel were performed (see Supplementary: Fig. 20, 21, 22 and Movie 7-8). As shown in the simulation Movies, the nBSC placed at the wall would minimally increase the local blood pressure (± 10%). This change in pressure is correlated to the average change of velocity ± 30%. Additional experiments (see Supplementary: Fig. 19 and Movie 3-6) were supplemented by placing the nBSC in a two-phase flow microfluidic channel to validate the simulations. Dulbecco's buffered saline (DPBS) was constantly pumped through two microfluidic channel inlets at a flow rate of 1 mm/s and additionally, blood was injected through the third channel inlet (see Supplementary: Fig. 19). This setup allows the visualization of the flow around the nBSC. As shown in the Supplementary: Movies 3-6, the flow profile of blood correlates with the simulations for the nBSC placed at the wall of the vessel.

In order for the nBSC to not cause any significant health risks, it should not block the blood vessel by more than 30 % [26]. Thus, by choosing a sufficiently large blood vessel diameter or by tuning the inner/outer diameter of the nBSC, one can efficiently implant the device without causing major vascular blockage (< 30%) or significant health risks.

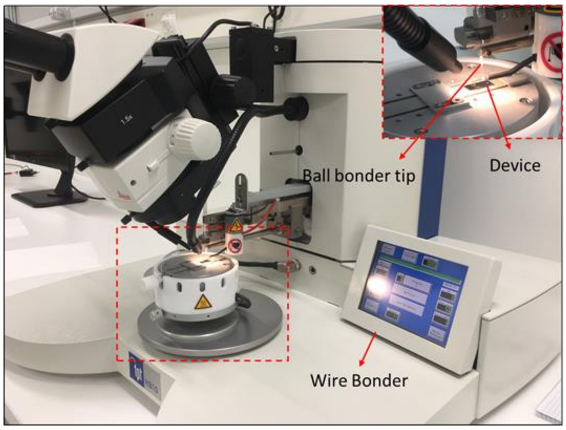

Supplementary Figure 23 | Self-protection measurement of nBSCs. Measurement setup for compression dependent analysis.

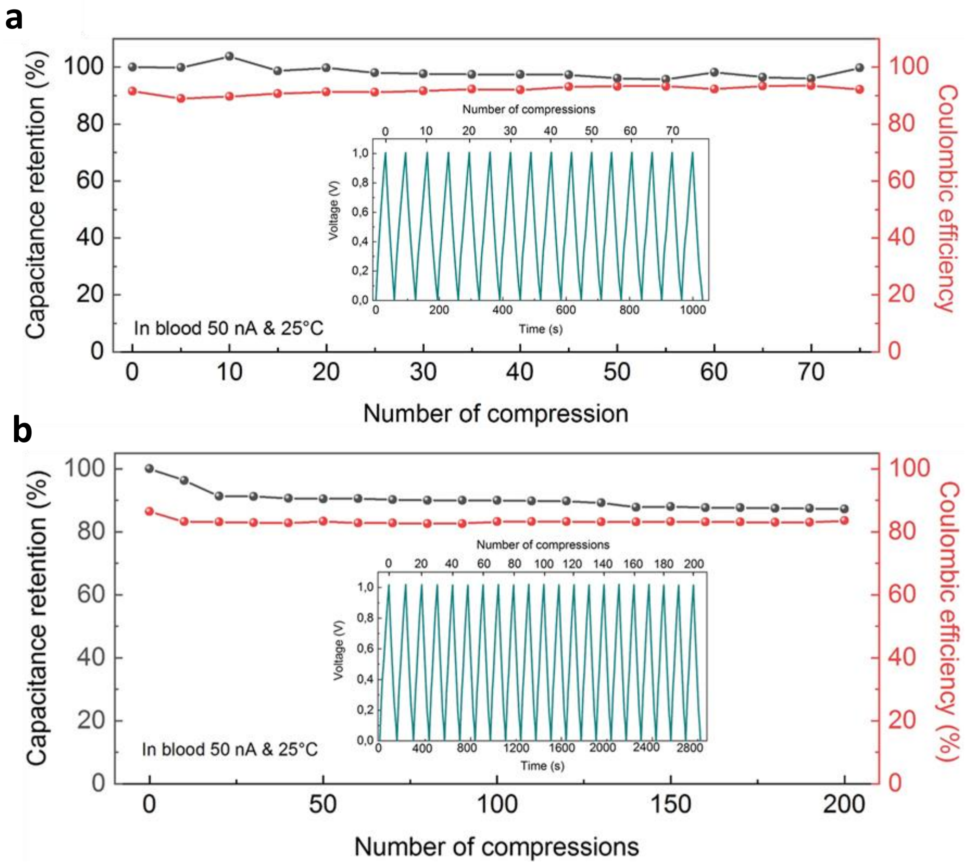

Supplementary Figure 24 | Self-protection measurement of nBSCs. a, Capacitance retention and coulombic efficiency of a nBSC under repeated compressions at 15 kPa over 75 cycles. Inset shows 75x GCD cycles (each GCD curve was measured after 5 compression cycles) of nBSC subjected to 15 kPa. **b,** Capacitance retention and coulombic efficiency of a nBSC under repeated compressions at 5 kPa over 200 cycles. Inset shows 200x GCD cycles (each GCD curve was measured after 10 compression cycles) of nBSC subjected to 5 kPa. (all measurements were performed at room temperature (25 °C) and static flow (0 ml/min).)

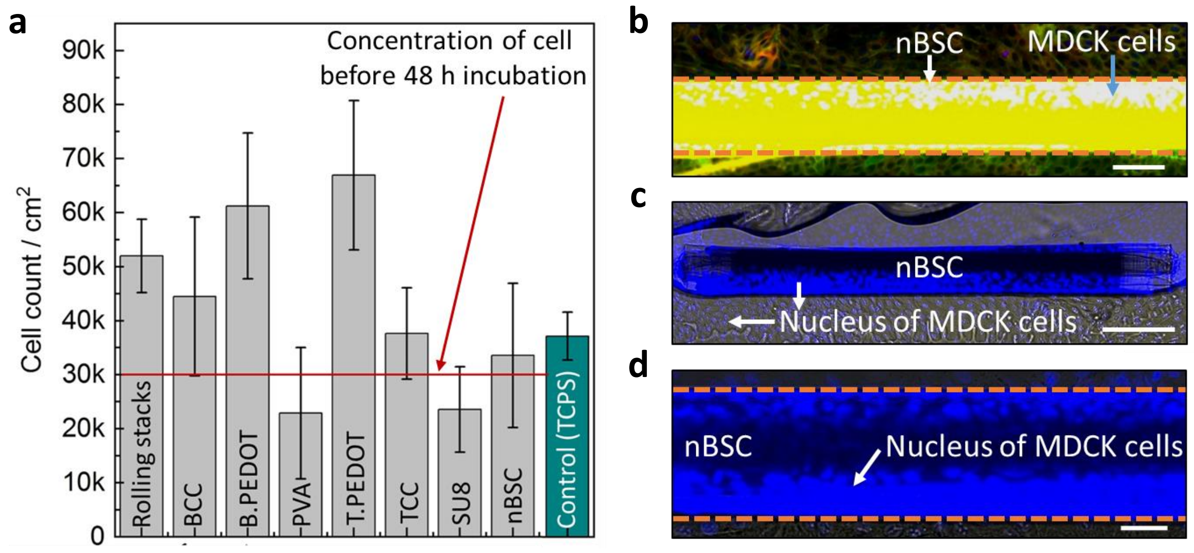

Supplementary Figure 25 | Effect of nBSCs on cytotoxicity. a, Cytotoxicity test: cell proliferation and count of madin-darby canine kidney (MDCK) cells incubated with all active materials of nBSC (BCC: Au bottom current collector, B.PEDOT: bottom PEDOT electrode, PVA: polyvinyl alcohol separator, T.PEDOT: top PEDOT electrode, TCC: top current collector, SU8 photoresist: passivation, nBSC: tubular nano-biosupercapacitor and TCPS: tissue culture polystyrene) over a period of 48 hours. All materials showed low to moderate cytotoxicity when compared to the control (TCPS). The red line represents initial concentration of MDCK-C7 cells in the culture media before 48 hours incubation. **b-d,** Fluorescent microscopic images to observe cell adhesion on different substrates with cell staining: green color: actin, blue color: DAPI and red color: vinculin. (Cells showed good proliferation and migrated inside the nBSC.) Scale bar, 50 µm **(b)**, 100 µm **(c)**, 25 µm **(d)**. (error bars represent variation in data of three measured devices.)

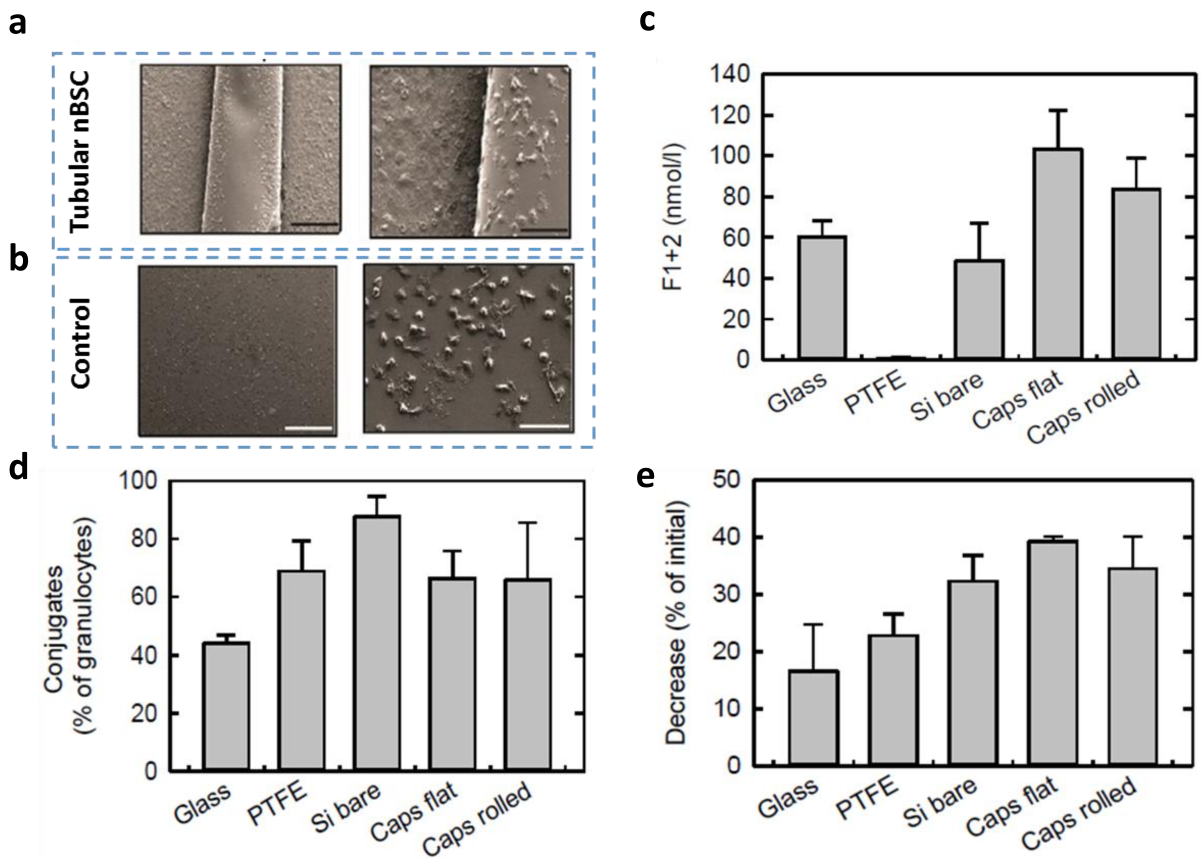

Supplementary Figure 26 | Coagulation and inflammation assay. a-b, SEM micrograph showing nBSC and control sample after incubation in low anticoagulated whole human blood over a period of two hours, showing less leukocytes and fibrin on the nBSC than on the carrier. Scale bars, 50 µm (left side of **a, b**), 5 µm (right side of **a, b**). **c,** Prothrombin fragment F1+2 as marker of plasmatic coagulation. **d,** Granulocyte-platelet conjugates as marker of platelet activation. **e,** complement fragment C5a as marker of inflammatory complement activation. (data presented as mean and standard deviation of six measured devices.)

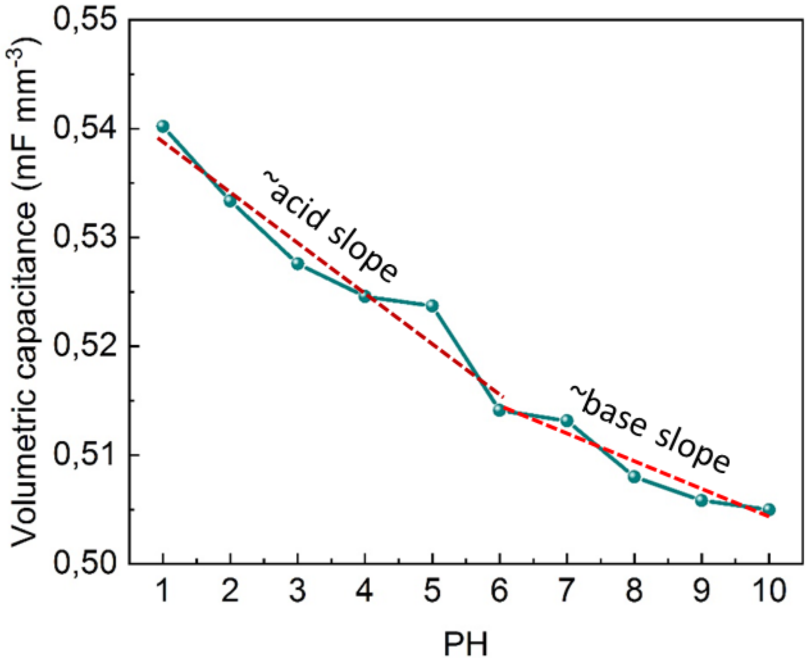

Supplementary Figure 27 | pH dependent volumetric capacitances of nBSCs. Volumetric capacitance as a function of electrolyte pH between 1 and 10. Red lines represent the relative change of volumetric capacitance per acid and base pH, respectively. All measurements were performed in artificial plasma at room temperature (25 °C) and under static conditions (0 ml/min).

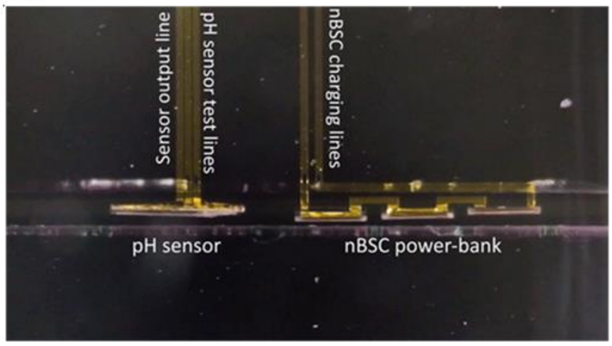

Supplementary Figure 28 | nBSC as a self-powered pH sensor. Optical image showing all components of self-powered nBSC based pH sensor integrated in microfluidic channel in electrolyte flow.

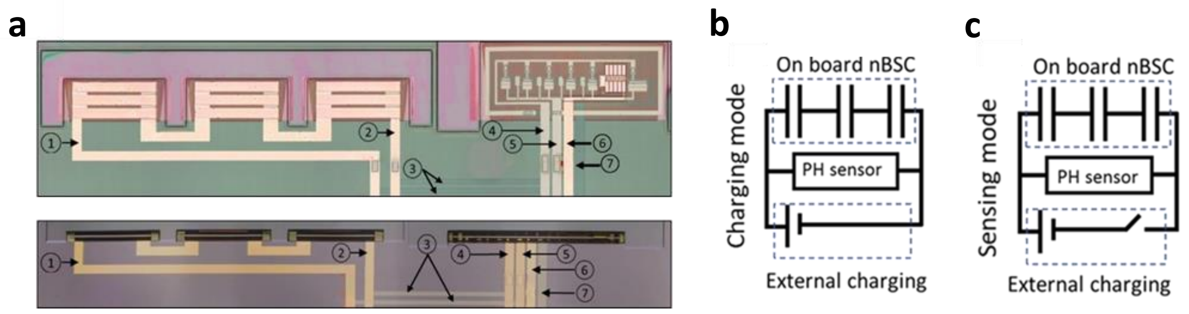

Supplementary Figure 29 | Measurement configuration of self-powered pH sensor. a, Optical image of nBSC integrated self-powered pH sensor (top) before rolling, and (bottom) after rolling with all its connection lines (1. External charging line (+Ve) connected to potentiostat; 2. External charging line (-Ve) connected to potentiostat; 3 . nBSC – pH sensor interconnect; 4. pH sensor power line (+Ve) connected to nBSC; 5. pH sensor groundline connected to nBSC; 6. Sensor unit test line; 7. pH sensor output to oscilloscope). **b,** Configuration of pH sensor in charging mode (external source charges the nBSC). **c,** Configuration of the pH sensor in measurement mode (nBSC powers the pH sensor).

Supplementary Note 8 Measurement configuration of self-powered pH sensor

As shown in Supplementary: Fig. 29a, the onboard nBSC is connected to an external potentiostat using charging lines (1,2) and the probe station setup described in Method section. The charging lines (1,2) are also connected to the power lines (4,5) using the onboard interconnect lines (3). The output line (7) is connected to the signal line and power line (5) to the ground of an oscilloscope using a microprobe station. After completing the setup, initially the onboard nBSCs are charged to 3V (charging mode Supplementary: Fig. 29b) using an external potentiostat through the charging line (1,2). Once the onboard nBSC is set to 3V, the external potentiostat is disconnected by removing the microprobe contacts from the charging lines (1,2). Thus, it allows the nBSC to discharge across the pH sensor through the interconnect (3), therefore powering the pH sensor and enabling autarkic operation. During the discharge phase of the nBSC the system is in sensing mode (see Supplementary: Fig. 29c). In this mode, the pH sensor is fed with different pH solution through a microfluidic channel and the corresponding output is measured through the output line (7). The pH sensor was operated in 3 stages:

State1: Discharged state (initial state of the device)

State2: Charging of the nBSCs at 1µA for ~ 30Sec

State3: Discharged of the nBSCs across the pH sensor (measurement phase)

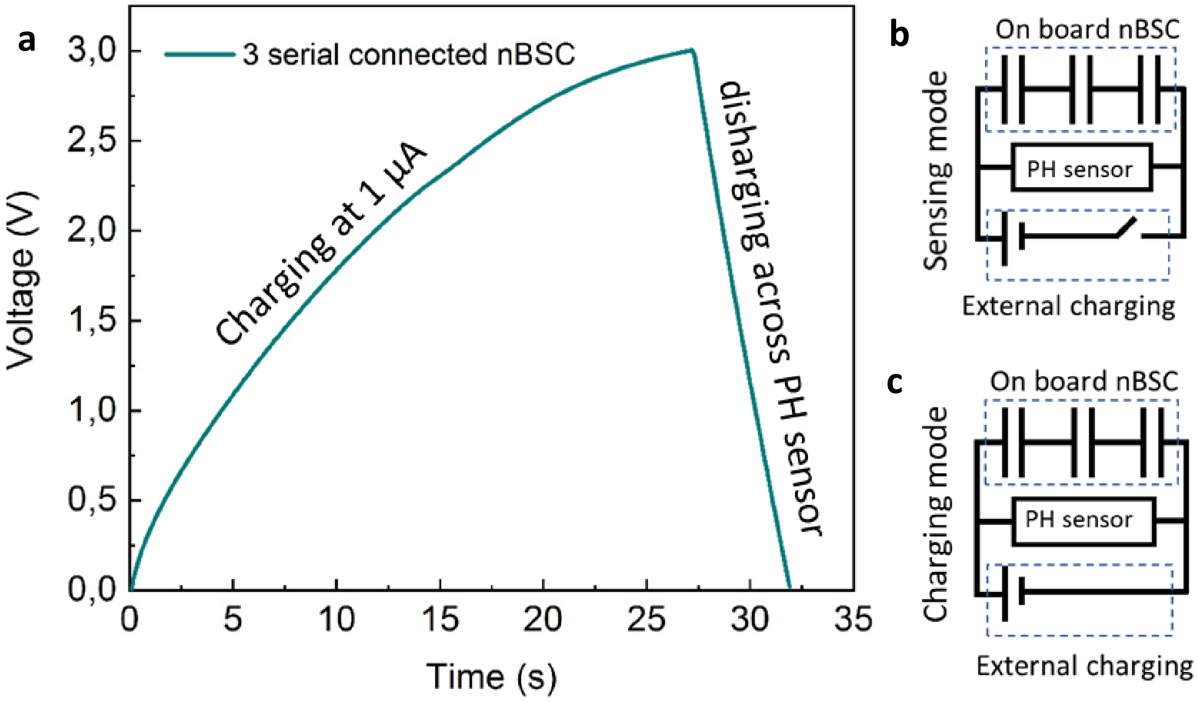

Supplementary Figure 30 | Charge/discharge characteristics of self-powered pH sensor. a, charge and discharge curve of three serially connected on-board nBSCs. Charging takes place at 1µA using an external power source followed by powering the pH sensor over ~ 5 s. **b,** Configuration of the pH sensor in the measurement mode (nBSC powers the pH sensor) **c,** Configuration of pH sensor in the charging mode (external source charges the nBSC).

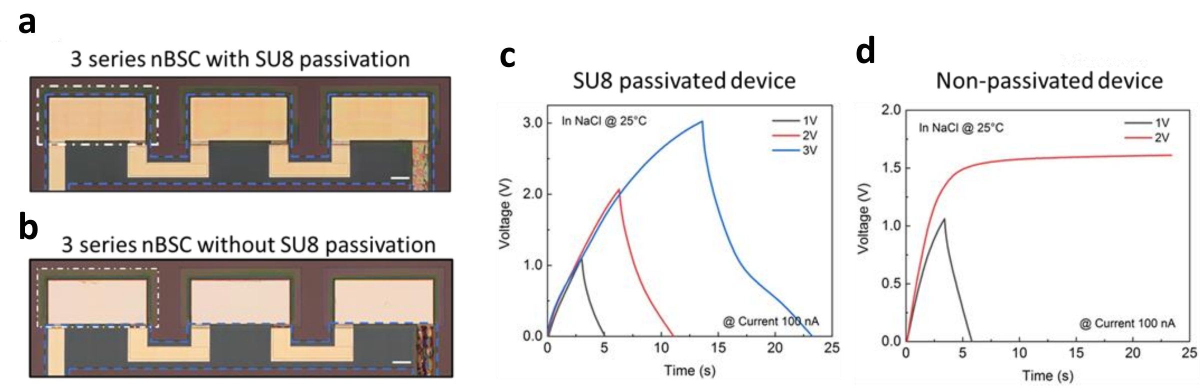

Supplementary Figure 31 | Effect of SU8 photoresist passivation on device and electrolyte stability. a, Microscope image of three nBSCs connected in series and passivated with SU8 photoresist. **b,** Microscope image of three nBSCs connected in series and without SU8 photoresist passivation (scale bar, 150μm; blue dotted line shows SU8 photoresist passivation area and the white dotted line shows PVA separator area). **c,** GCD curve of SU8 photoresist passivated device charged to 1, 2 and 3V at 100 nA. **d,** GCD curve of non-passivated device charged to 1 and 1.5V at 100 nA.

Supplementary Note 9 Effect of SU8 photoresist passivation on device and electrolyte stability

The three BSCs connected in series can charge up to 3V while maintaining contact with the blood due to the quasi-electrical isolation provided by the SU8 photoresist passivation and the PVA hydrogel separator. The SU8 photoresist passivates the complete nBSC except for a small portion of the electrically neutral PVA hydrogel separator. The SU8 photoresist passivation includes the current collectors, electrodes, and interconnects (as indicated by the blue dotted line in Supplementary: Fig. 31a) protecting the nBSC from all possible electrical short-circuit paths through the electrolyte. Additionally, the electrochemical impedance of the PVA hydrogel separator at the edge of the nBSC tube is extremely high. This property avoids any ionic short-circuit paths while the PVA absorbs and supplies the electrolyte to the PEDOT electrode surface for electrochemical charge storage. In order to prove this, three nBSCs connected in series were prepared with and without SU8 photoresist passivation as shown in Supplementary: Fig. 31a-b. These series-connected nBSCs were charged to 3V at 100 nA. As shown in Supplementary: Fig. 31c-d, the nBSC devices which are passivated reach the charging voltage of 3V. In contrast, the non-passivated device could only reach a maximum single cell potential of 1.5V at the same charging current showing that electronic and ionic isolated SU8 photoresist devices can be connected in series and charge to high voltage while sharing the same electrolyte.

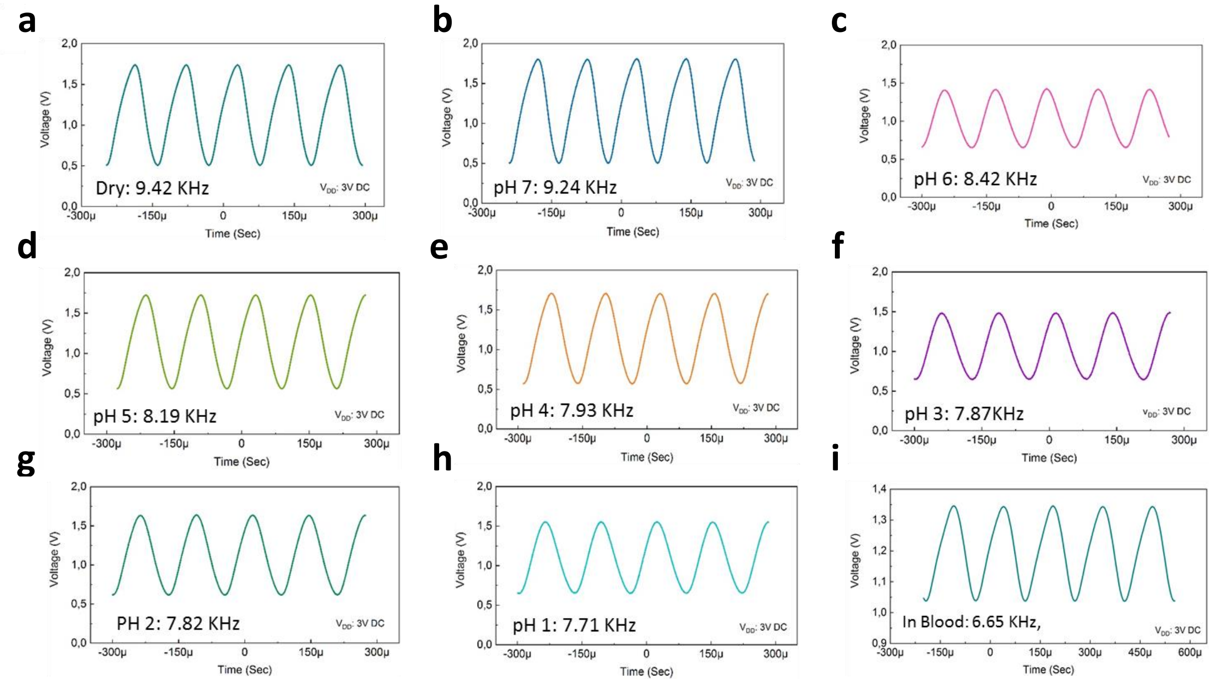

Supplementary Figure 32 | Frequency modulation of nBSC based sensor as a function of electrolyte pH. a-i, Output of five-stage oscillator at 3V DC input in **(a)** dry state, **(b)** electrolyte pH 7, **(c)** electrolyte pH 6, **(d)** electrolyte pH 5, **(e)** electrolyte pH 4, **(f)** electrolyte pH 3, **(g)** electrolyte pH 2, **(h)** electrolyte pH 1 (measurements were performed in artificial plasma), **(i)** blood electrolyte (pH 6~7, frequency differs compared to artificial plasma at pH 6 due to increased capacitance via bioenhancement effect). All measurements were performed at room temperature (25 °C) and uniform flow (2 ml/min).

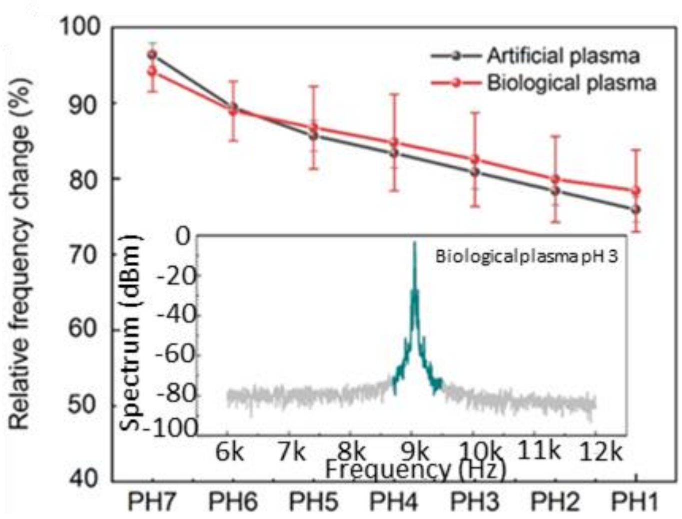

Supplementary Figure 33 | nBSC as a self-powered pH sensor. Relative frequency change of nBSC as a function of electrolyte pH of biological and artificial plasma. Inset figure: Frequency spectral response of the nBSC based pH sensor at pH 3 of blood plasma.

Supplementary Note 10 nBSC as a self-powered pH sensor

Complex biological fluids like blood indeed have several other components that would influence the pH sensor's selectivity and sensitivity. Additional experiments by subjecting the pH sensor into blood plasma were performed. The pH of blood plasma was altered by adding and adjusting the concentration of citric acid to achieve a blood plasma pH from 1 to 7. The self-powered pH sensor was used to sense the pH of the altered blood plasma (see Supplementary: Fig. 28). The sensor showed a relative frequency change of -2.7 E-2 ± 2.0 E-2 per pH and yields larger error bars in blood plasma compared to the relative frequency change of -2.4 E-2 ± 1.9 E-4 per pH in artificial plasma (see Supplementary: Fig. 33). The larger error bar reflects the complex activity of the enzymes present in blood plasma. This is due to the relative broadening of the pH spectrum (see Supplementary: Fig. 33 inset) in blood plasma compared to artificial plasma (Figure 4d). As observed in the frequency spectral response of the nBSC (see Supplementary: Fig. 33 inset), broader and several overlapping frequency peaks are typical for the response in blood plasma compared to the narrow frequency spectral response of the nBSC in artificial plasma (Figure 4d). This indicates that the complex composition of the biological fluid consisting of glucose, redox enzymes, and other chemicals influences the capacitance of the nBSC and significantly affects the sensitivity.

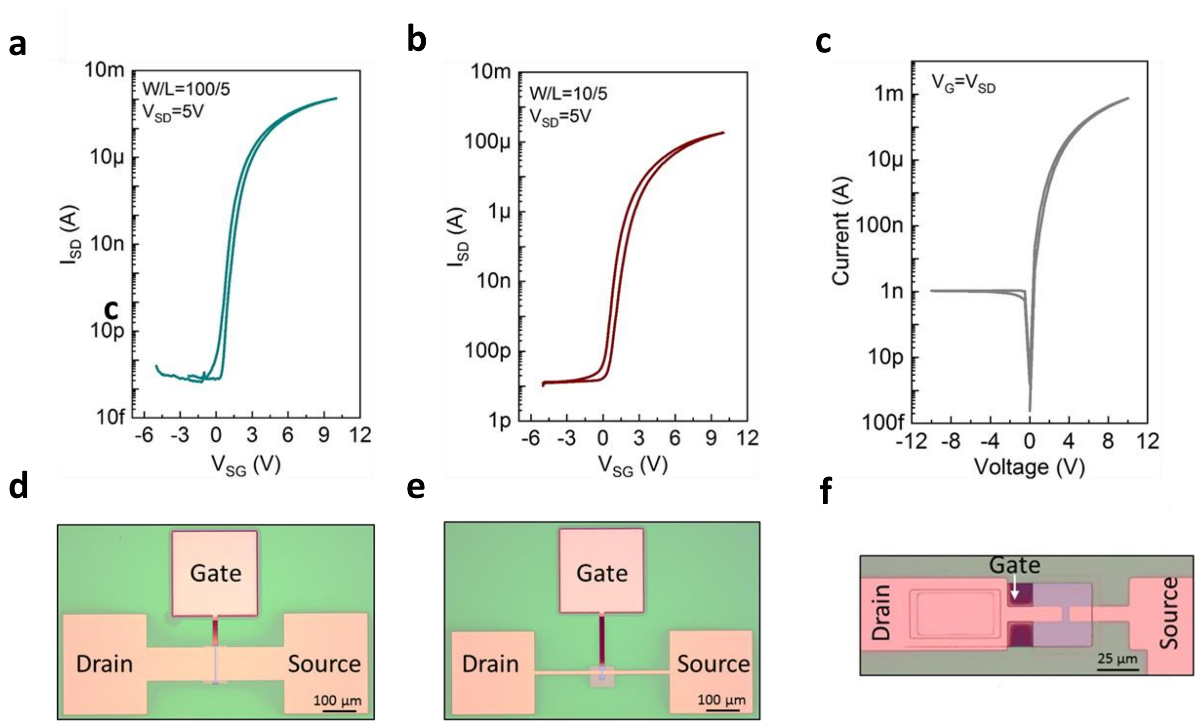

Supplementary Figure 34 | Performance of fundamental circuit building blocks: the transistor. a, Transfer characteristic of active transistor with W=100 µm, L=5 µm at V_{SD}=5V. **b,** Transfer characteristics of load transistor with W=10 µm, L=5 µm at V_{SD}=5V. **c,** Transistor output in load configuration. **d-f,** Microscopic images of **(d)** active transistor, **(e)** load transistor, and **(f)** transistor in load configuration.

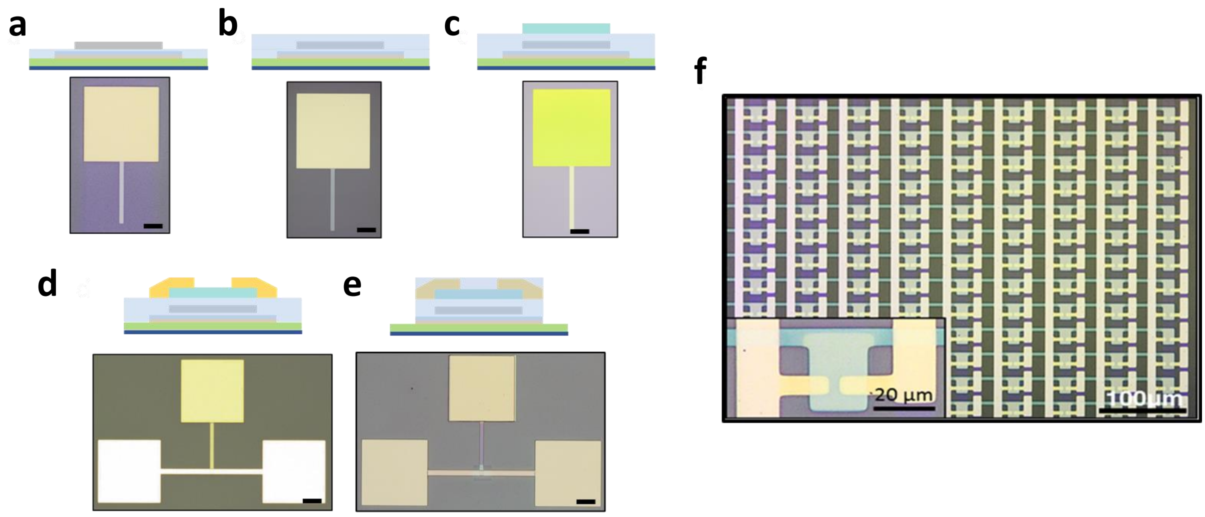

Supplementary Figure 35 | Schematic illustration and optical images of thin-film-transistor (TFT) fabrication. a, Gate electrode, Cr. **b,** Gate-dielectric layer, $HfO_2/Al_2O_3/HfO_2$. **c,** Active semiconductor layer, ZnO. **d,** Source and drain layer, Ti/Au. **e,** Passivation layer, PI/SiO_2. Scale bar, 30 µm **(a-c)** and 60 µm **(d, e). f,** Microscopic image of TFT array.

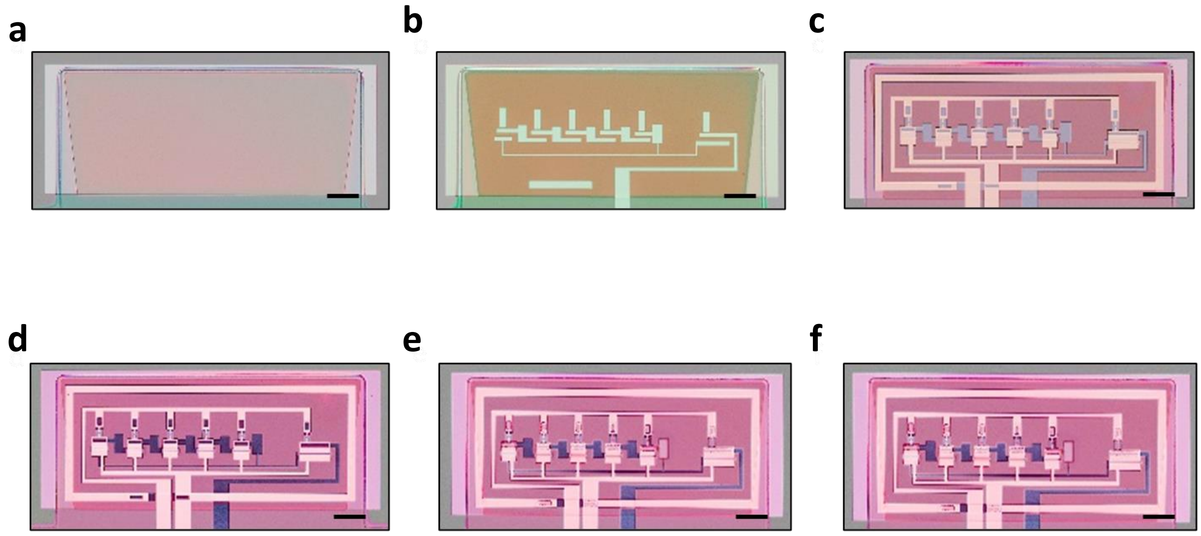

Supplementary Figure 36 | Fabrication protocol of five-stage ring oscillator for pH sensor. a, Polymeric rolling stack. **b,** Gate electrode, Cr. **c,** Source and drain electrode, Ti/Au. **d,** Active semiconductor etching, ZnO in solution of 0.033 part (v/v) of phosphoric acid, 0.066 part (v/v) of citric acid and 10 part (v/v) of DI water. **e,** Gate dielectric etching, HfO2 / Al2 O3 / HfO2 in 10% buffer HF. **f,** Via filling, Ti/Au. Scale bar, 200 µm **(a-f).**

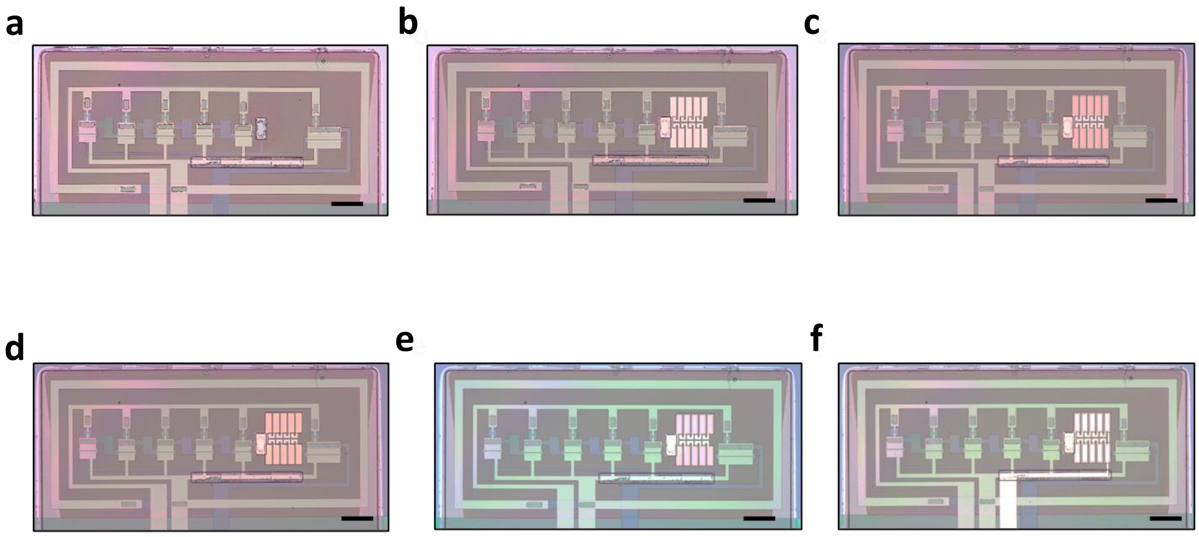

Supplementary Figure 37 | Fabrication protocol of integrating nBSC as a pH sensor. a, Passivation of five-stage ring-oscillator. **b,** Bottom current collector, Cr/Au. **c,** Bottom electrode, PEDOT:PSS. **d,** Proton exchange separator, photo-patterned PVA. **e,** Top electrode, PEDOT:PSS. **f,** Top current collector, Cr/Au. Scale bar, 200 µm **(a-f).**

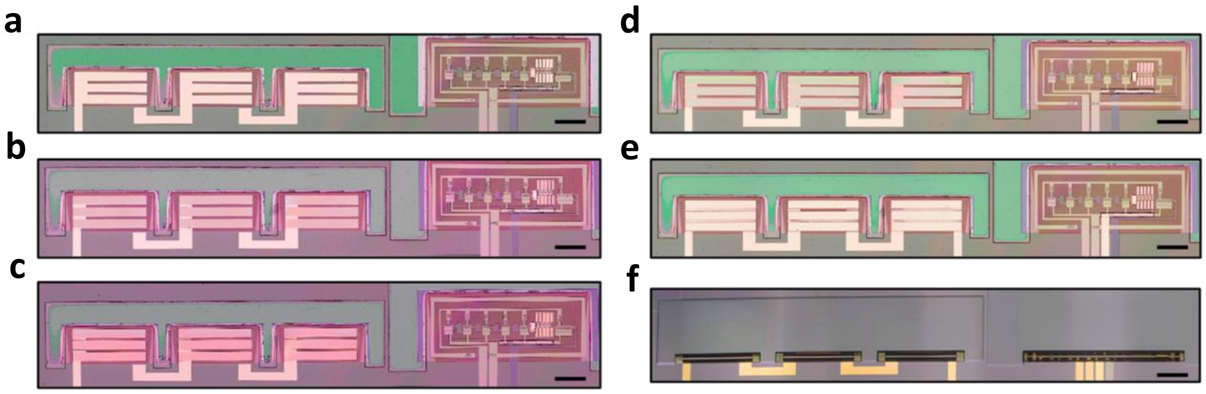

Supplementary Figure 38 | Fabrication protocol of nBSC based on-board energy storage unit for pH sensor. a, Bottom current collector, Cr/Au. **b,** Bottom electrode, PEDOT:PSS. **c,** Proton exchange separator, photo-patterned PVA. **d,** Top electrode, PEDOT:PSS. **e,** Top current collector, Cr/Au. **f,** Tubular nBSC based pH sensor after rolling, 200 µm **(a-f). g,** Configuration of pH sensor in charging mode. **h,** Configuration of pH sensor in measurement mode.

Ingredients	Ingredients concentration. g/dm^3 distilled water
NaCl	6.800
$CaCl_2$	0.200
KCl	0.400
$MgSO_4$	0.100
$NaHCO_3$	2.200
Na_2HPO_4	0.126
NaH_2PO_4	0.026

Supplementary Table 1 | Chemical composition of artificial plasma[27].

Supplementary References

1. Karnaushenko, D. *et al.* Self-assembled on-chip-integrated giant magneto-impedance sensorics. *Adv Mat* **27,** 6582 (2015).

2. Karnaushenko, D. *et al.* Biomimetic microelectronics for regenerative neuronal cuff implants. *Adv Mat* **27,** 6797 (2015).

3. Jensen, B. E. B, Dávila, I. & Zelikin, A. N. Poly(vinyl alcohol) physical hydrogels: Matrix-mediated drug delivery using spontaneously eroding substrate. *J Phys Chem B* **26,** 120 (2016).

4. Ahmed, A. A. A., Al-Hussam, A. M., Abdulwahab, A. M. & Ahmed, A. N. A. A. The impact of sodium chloride as dopant on optical and electrical properties of polyvinyl alcohol. *AIMS Mater Sci* **5,** 533 (2018).

5. Sanyal, S., Bhui, U. K., Kumar, S. S. & Balaga, D. Designing injection water for enhancing oil recovery from kaolinite laden hydrocarbon reservoirs: A spectroscopic approach for understanding molecular level interaction during saline water flooding. *Energy Fuels* **31,** 11627 (2017).

6. Pang, J. *et al.* Green aqueous biphasic systems containing deep eutectic solvents and sodium salts for the extraction of protein. *RSC Adv* **7,** 49361 (2017).

7. Hu, Y. J., Yang, Y. O. & Zhang, Y. Affinity and specificity of ciprofloxacin-bovine serum albumin interactions: Spectroscopic approach. *Protein J* **29,** 234 (2010).

8. Shilova, O. N., Shilov, E. S. & Deyev, S. M. The effect of trypan blue treatment on autofluorescence of fixed cells. *Cytom A* **91,** 917 (2017).

9. Tuchin, V. V., Zhestkov, D. M., Bashkatov, A. N. & Genina, E. A. Theoretical study of immersion optical clearing of blood in vessels at local hemolysis. *Opt Express* **12,** 2966 (2004).

10. Mallya, M. *et al.* Absorption spectroscopy for the estimation of glycated hemoglobin (HbA1c) for the diagnosis and management of diabetes mellitus: A pilot study. *Photomed Laser Surg* **31,** 219 (2013).

11. Feldschuh, J. & Enson, Y. Prediction of the normal blood volume: Relation of blood volume to body habitus. *Circulation* **56,** 4 (1977).

12. De Buck, S. S. et al. Prediction of human pharmacokinetics using physiologically based modeling: A retrospective analysis of 26 clinically tested drugs. *Drug Metab Dispos* **35,** 10 (2007).

13. Biological Responses to Metal Implants – FDA, September (2019).

14. Davidovsky, A.G. The aging, biodegradation and toxic effects of the polymer biomedical implants. *Dental Practice* **869,** 259 (2020).

15. Eliaz, N. Corrosion of metallic biomaterials: A review. *Materials (Basel)* **12,** 407 (2019).

16. Manivasagam, G., Dhinasekaran, D., & Rajamanickam, A. Biomedical implants: Corrosion and its prevention - A review. *Recent Pat Corros Sci* **2,** 40, (2010).

17. Rehman, M. U., Jawaid, P., Uchiyama, H. & Kondo, T. Comparison of free radicals formation induced by cold atmospheric plasma, ultrasound, and ionizing radiation. *Arch Biochem Biophys* **605,** 19 (2016).

18. Stoffels, E. *et al.* Mass spectrometric detection of short-living radicals produced by a plasma needle. *Plasma Sources Sci Technol* **16,** 549 (2007).

19. Nevers, D. R., Brushett, F. R. & Wheeler, D. R. Engineering radical polymer electrodes for electrochemical energy storage. *J Power Sources* **352,** 226 (2017).

20. Nishide, H. & Suga, T. Organic radical battery. *Electrochem Soc Interface* **14,** 32 (2005).

21. Friebe, C., Lex-Balducci, A. & Schubert, U. S. Sustainable energy storage: recent trends and developments toward fully organic batteries. *ChemSusChem* **12,** 4093 (2019).

22. Casado, N. *et al.* PEDOT radical polymer with synergetic redox and electrical properties. *ACS Macro Lett* **5,** 59 (2016).

23. Jha, N., Ryu, J. J., Choi, E. H. & Kaushik, N. K. Generation and role of reactive oxygen and nitrogen species induced by plasma, lasers, chemical agents, and other systems in dentistry. *Oxid Med Cell Longev* (2017).

24. Halliwell, B. & Gutteridge, J. M. C. Oxygen toxicity, oxygen radicals, transition metals and disease. *BioChem J* **219,** 1 (1984).

25. Rahman, A. *et al.* Strand scission in DNA induced by dietary flavonoids: role of Cu (I) and oxygen free radicals and biological consequences of scission. *Mol Cell Biochem* **111,** 3 (1992).

26. McMains, V. and Nelson, L. Updated classification system captures many more people at risk for heart attack. Available from: https://www.hopkinsmedicine.org/news/media/releases/updated_classification_system_captures_many_more_people_at_risk_for_heart_attack (2017).

27. Kokubo, T. *et al.* Solutions able to reproduce *in vivo* surface-structure changes in bioactive glass-ceramic A-W. *J Biomed Mater Res* **24,** 721 (1990).
