# Supplementary material for: Nano-biosupercapacitors enable autarkic sensor operation in blood
Source: Nat Commun. 2021 Aug 23;12:4967. doi: 10.1038/s41467-021-24863-6 (PMC8382768; doi:10.1038/s41467-021-24863-6)
Supplement: Supplementary file 3 — Description of Additional Supplementary Files [file 41467_2021_24863_MOESM3_ESM.pdf]

Description of Additional Supplementary Files

Supplementary Movie 1:

Effect of lacking passivation on planar nBSC.

Supplementary Movie 2:

Effect of multiple GCD cycling on blood plasma plus 0.5% redox dye.

Supplementary Movie 3:

Experimental setup - blood flow profile

Supplementary Movie 4:

Blood Flow Profile at 0.05 mms-1

Supplementary Movie 5:

Blood Flow Profile at 0.5 mms-1

Supplementary Movie 6:

Blood Flow Profile at 1 mms-1

Supplementary Movie 7:

Simulation of blood flow velocity profile as a function of nBSC tube diameter.

Supplementary Movie 8:

Simulation of blood flow velocity profile as a function of nBSC wall thickness.
